# Analysis of a fractional-order model for dengue transmission dynamics with quarantine and vaccination measures

**DOI:** 10.1038/s41598-024-62767-9

**Published:** 2024-05-25

**Authors:** Muhammad Usman, Mujahid Abbas, Safeer Hussain Khan, Andrew Omame

**Affiliations:** 1 Abdus Salam School of Mathematical Sciences, Government College University Katchery Road, Lahore, 54000 Pakistan; 2 Department of Mathematics, Government College University Katchery Road, Lahore, 54000 Pakistan; 3 Department of Medical Research, China Medical University Hospital, China Medical University, Taichung, 40402 Taiwan; 4 Department of Mathematics and Statistics, North Carolina A &T State University, Greensboro, NC 27411 USA; 5 Department of Mathematics, Federal University of Technology, Owerri, Nigeria

**Keywords:** Dengue, Strains, Mathematical model, Existence and uniqueness, Stability, Reproduction number, Nonstandard finite difference scheme, Applied mathematics, Mathematics and computing

## Abstract

A comprehensive mathematical model is proposed to study two strains of dengue virus with saturated incidence rates and quarantine measures. Imperfect dengue vaccination is also assumed in the model. Existence, uniqueness and stability of the proposed model are proved using the results from fixed point and degree theory. Additionally, well constructed Lyapunov function candidates are also applied to prove the global stability of infection-free equilibria. It is also demonstrated that the model is generalized Ulam-Hyers stable under some appropriate conditions. The model is fitted to the real data of dengue epidemic taken from the city of Espirito Santo in Brazil. For the approximate solution of the model, a non-standard finite difference(NSFD) approach is applied. Sensitivity analysis is also carried out to show the influence of different parameters involved in the model. The behaviour of the NSFD is also assessed under different denominator functions and it is observed that the choice of the denominator function could influence the solution trajectories. Different scenario analysis are also assessed when the reproduction number is below or above one. Furthermore, simulations are also presented to assess the epidemiological impact of dengue vaccination and quarantine measures for infected individuals.

## Introduction

The virus that causes dengue fever is spread mostly by female Aedes mosquitoes, predominantly Aedes aegypti mosquitoes, and secondarily Aedes albopictus mosquitoes. Through mosquito bites, the dengue virus can infect humans and cause mild or severe sickness or death in some cases^1^. The areas with a high Aedes mosquito population are most likely to experience a dengue outbreak. The disease is primarily spread when an adult female Aedes mosquito bites a person carrying the virus, catching it and then passing it on to an uninfected person. However, there are other less common ways to spread the virus including through breastfeeding and pregnancy and in extremely rare instances, through organ transplantation^2^. Four Dengue serotypes (Dengue1-4) have spread quickly inside nations and across continents, resulting in epidemics and severe dengue fever, hyperendemicity of numerous Dengue serotypes in tropical nations, and autochthonous transmission in Europe and the USA^3,4^. In the past, there was no specific treatments for dengue. The only possibility to control the disease was to control the vectors, which was very difficult. In 2015, a new vaccine for Dengue virus (*Dengvaxia* by Sanofi Pasteur) has been released^5^. *Dengvaxia* is a tetravalent vaccine whose efficacy varies by serotypes ( 54.7% for serotype 1, 43.0% for serotype 2, 71.6% for serotype 3 and 76.9% for serotype 4.)^6^.

Mathematical models using the classical integer-order derivative have been developed in studying the dynamics of infectious diseases^7–18^. In particular, Ferguson et al.^14^ employed a PDE model that takes prior infection history into account. Using an agent-based dengue model. Hladish et al.^15^ investigated the effects of several vaccine scenarios on dengue transmission dynamics in Yucatan, Mexico. For the purpose of examining the effects of immunisation against the four dengue serotypes, Coudeville and Garnett^16^ considered an age-structured compartmental model. A similar (age-stratified) model was employed by Rodriguez-Barraquer et al.^17^ to assess the effectiveness of a vaccination that is only partially effective against three of the four dengue serotypes. Using compartmental and agent-based modelling techniques, Chao et al.^18^ demonstrated that a dengue vaccine with efficacy ranging from 70% to 90% against all four dengue serotypes has the potential to reduce the frequency and magnitude of dengue epidemics significantly in the short and medium terms.

These models, due to the integer nature of the derivative constitute certain limitations. Different fractional operators relying on power-law^19^, exponential^20^, generalized Mittag-Leffler^21^ and other forms of kernels have emerged and their applications to modelling biological processes have gained much attraction in recent times. The fractional order operators have some advantages over classical order operator such as memory effect and better performance. Mathematical models using the fractional order operators have been successfully applied in investigating the dynamics of the infectious diseases^22–27^. In particular, Fatmawati^27^ studied the dengue dynamics with fractal-factional Caputo-Fabrizio operator and employed real statistical data of dengue infection cases of East Java, Indonesia, from 2018 and parameterized the dengue model.

The Caputo fractional operator with a singular kernel offers advantages in modeling disease transmissions by providing a more flexible framework that can capture memory effects, non-local behavior, and complex dynamics. Memory effects means that it accounts for the history of the system. In disease transmission models, this can be particularly useful for capturing the impact of past infections, immunity, or interventions on the current state of the population. Unlike classical derivatives, fractional derivatives are non-local operators showing that the behavior of the system does not depend on the neighbourhood of a particular point but depends on its history over a range of time, which can be crucial for modeling the spread of infectious diseases where past interactions can influence future outcomes. The non standard finite difference (NSFD) scheme has a couple of favourable properties. It is explicit and due to its construction it reproduces important properties of the solution, like the number and location of fixed-points, the positivity, accuracy, stability and certain conservation laws. It offer valuable tools for numerical simulation and analysis across a wide range of scientific and engineering disciplines, where accurate and efficient approximation of model solutions is essential for understanding and predicting complex phenomena.

In this study, based on Caputo fractional operator, a comprehensive model for two strains of dengue is proposed, and validated using data from Brazilian state of Espirito Santo. Given that both vaccines have varying levels of efficacy, the suggested model also assumes separate immunization for strains-1 and strain-2. We have also included two co-infection compartments, which have not been considered in the existing models, for possible disease states, including exposed, asymptomatic, and symptomatic infections. We established the conditions for existence, uniqueness and stability of the model. In addition, we applied the nonstandard finite difference (NSFD) scheme to obtain approximate solution of the model. Then impact of different denominator functions on the approximated solution is also presented. To the best of our knowledge, the proposed model studied in this paper is novel and appropriate to study the co-circulation of two dengue strains using fractional calculus tools.

The paper is organized as follows: The model is formulated in “Model formulation”. The rigorous analysis of the sub-model and the full model is given in “Analysis of the sub-models” and “Analysis of the complete model”. Existence and uniqueness are proved in the “Existence, uniqueness and Ulam–Hyers stability of the complete model”. The Ulam-Hyers stability is presented in “Ulam–Hyers stability”. The model solution is approximated with the help of non standard finite difference scheme in “Nonstandard finite difference scheme”. The model fitting and the numerical assessments are given in “Model fitting and numerical assessment”. Finally “Conclusion” contains the concluding remark and some future directions.

### Preliminaries

#### Definition 1.1

^19^ The Caputo fractional derivative of a function *f* of order $\sigma \in (0,1)$ is defined by

1 $$\begin{aligned} ^{\tiny \textsc {C}}D_{t}^{\sigma }f(t)=\frac{1}{\Gamma (n-\sigma )}\int _{0}^{t}(t-\wp )^{n-\sigma -1}f^{(n)}(\wp )d\wp , \end{aligned}$$

where, $n = [\sigma ]+1$ and $\Gamma$ stands for the Gamma function.

#### Definition 1.2

^19^ The Riemann–Liouville fractional integral of a function *f* of order $\sigma \in (0,1)$ is defined by

2 $$\begin{aligned} ^{\tiny \textsc {C}}I_{t}^{\sigma }f(t)=\frac{1}{\Gamma (\sigma )} \int _{0}^{t}(t-\wp )^{\sigma -1}f(\wp )d\wp ,\hspace{0.8cm}t>0, \end{aligned}$$

#### Lemma 1.1

^19^ The Laplace transform of Caputo fractional derivative is given by

3 $$\begin{aligned} {\mathcal {L}}\left\{ ^{\tiny \textsc {C}} D^\sigma _t f(t)\right\} = s^\sigma {\mathcal {L}} \{f(t)\} -s^{\sigma -1}f(0), \quad 0<\sigma <1, \end{aligned}$$

where ${\mathcal {L}}$ is the Laplace transform operator.

We now recall the following definitions from^28^.

#### Definition 1.3

The Kuratowski measure of non-compactness $\mu : {\mathbb {B}} \rightarrow [0, \infty )$ is defined as:

$$\begin{aligned} \mu (\Omega )=\inf \{d>0: \Omega \in {\mathbb {B}}~\text {admits a finite cover by sets of diameter}\leqslant d\}, \end{aligned}$$

where ${\mathbb {B}}$ denotes the family of all bounded subsets of *E*.

Recall that the Kuratowski measure $\mu$ has the property that $\mu (\Omega )=0$ iff $\Omega$ is relatively compact.

#### Definition 1.4

A continuous and bounded function $T: S \rightarrow E$ is said to be $\mu$-Lipschitz if there exist $k \ge 0$ such that

$$\begin{aligned} \mu (T(\Omega _{0})) \le k \mu (\Omega _{0}) \end{aligned}$$

for all bounded subsets of $S \subseteq E.$

#### Definition 1.5

The mapping *T* is said to be $\mu$-condensing if

$$\begin{aligned} \mu (T(\Omega _{0})) < \mu (\Omega _{0}) \end{aligned}$$

for all bounded subset of S.

#### Lemma 1.2

If A and B are $$\mu $$-Lipschitz map with constant *k* and $$k^{'} $$ respectively then A+B is also $$\mu $$-Lipschitz with constant $$k+k^{'}.$$

#### Lemma 1.3

If T is a compact map, then T is $\mu$-Lipschitz with constant 0.

#### Lemma 1.4

If T is Lipschitz map with with constant k, then T is $\mu$-Lipschitz map with the same constant.

#### Theorem 1.1

Let $T: E \rightarrow E$ be $\mu$-condensing and

$$\begin{aligned} \Theta =\{\zeta \in E: \exists \quad \lambda \in [0,1] \quad \text{ such } \text{ that } \zeta =\lambda T \zeta \} \text{. } \end{aligned}$$

If $\Theta$ is a bounded set in *E*, that is there exists $r>0$ such that $\Theta \subset B_r(0)$, then the degree

$$\begin{aligned} {\mathbb {D}}\left( I-\lambda T, B_r(0), 0\right) =1, \quad \forall \quad \lambda \in [0,1]. \end{aligned}$$

Consequently, *T* has at least one fixed point and the set of the fixed points of *T* lies in $B_r(0)$.

## Model formulation

To formulate the model, the human population at a given time *t* is denoted by *N*(*t*) and is subdivided into following classes: vulnerable or uninfected persons $S_h (t)$, individuals vaccinated against Dengue $V_{h}(t)$, individuals exposed to Dengue strain 1 and strain 2 $E_{h1} (t),E_{h2} (t),$ respectively, individuals exposed for co-infection $E_{h12}(t)$, Individuals infected with strain 1, strain 2 and both disease (Asymptomatic stage) $A_{h1}(t),A_{h2}(t), A_{h12}(t),$ respectively, Individuals infected with strain 1, strain 2 and both disease (Symptomatic stage) $I_{h1}(t),I_{h2}(t), I_{h12}(t),$ respectively, quarantine individuals suffering with strain 1, strain 2 and both disease $Q_{h1}(t),Q_{h2}(t), Q_{h12}(t),$ respectively, recovered individuals from strain 1, strain 2 and co-infection are $R_{h1}(t),R_{h2}(t),R_{h12}(t),$ respectively. The vector population is divided into: Susceptible vectors $S_v(t)$, Vectors exposed to strain 1 and strain 2 $E_{v1}(t), E_{v2}(t),$ respectively, vectors infected with strain 1 and strain 2 $I_{v1}(t), I_{v2}(t),$ respectively.

Based on the established knowledge about the epidemiology of dengue serotypes^29–31^, the proposed model has the following assumptions:

- Susceptible individuals can get infections with dengue strain-i from infected vectors at the rate of $\frac{\beta _{hi}^{\sigma } I_{vi}}{1+\alpha _{i}^{\sigma }I_{vi}}$ for $i=1,2$.
- The saturated form of incidence $\frac{\beta _{hi}^{\sigma } I_{vi}}{1+\alpha _{i}^{\sigma }I_{vi}}$ for $i=1,2$ is adopted in this model. Basically we are adding the parameters $\alpha _i^{\sigma }$ to add some control in the transmission due to the crowding effect and inhabitation effect and behavioral change by the susceptible individuals. This has been used in some epidemiological models^32–34^.
- Susceptible individuals are vaccinated at the rate $\psi ^{\sigma }$. The dengue vaccine is assumed to have efficacy of $\phi _i^{\sigma }$ against strain i.
- Individuals infected with either strain 1 or strain 2 can get infected with the other strain.
- Natural death rate is assumed to be $\mu _h^{\sigma }$ for all human compartments.
- Symptomatic individuals are quarantined at the rate $\eta _{i}^{\sigma }$.
- Removal of vectors from the population is assumed at the rate $\mu _v^{\sigma }$.
- The recovered individuals can lose their immunity and return to the susceptible state at the rate $\delta _{hi}^{\sigma }.$

The model’s parameters are described in Table 1 whereas the system’s equations are presented in (4).

**Table 1:** Model (4) parameters’ description.

Parameter	Description	Value	References
$\beta _{h1}^{\sigma }$	Transmission rate for dengue Strain 1	4.3892e-13 $day^{-1}$	Fitted
$\beta _{h2}^{\sigma }$	Transmission rate for dengue Strain 2	$5.1160e-07 \times 10^{-6}$ day$^{-1}$	Fitted
$\theta _{a1}^{\sigma }$	Mortality rates for Asymptomatic Individuals of strain 1	0.0406 day$^{-1}$	Fitted
$\theta _{a2}^{\sigma }$	Mortality rates for Asymptomatic Individuals of strain 2	4.2968e−07 day$^{-1}$	Fitted
$\psi ^{\sigma }$	Vaccination rate	7.0002e-05 $day^{-1}$	Fitted
$\zeta _{a1}^{\sigma }$	Recovery rates from Asymptomatic individuals of strain 1	0.0544 day$^{-1}$	Fitted
$\zeta _{a2}^{\sigma }$	Recovery rates from Asymptomatic individuals of strain 2	3.9726e−07 day$^{-1}$	Fitted
$\alpha _1^{\sigma }, \alpha _2^{\sigma }$	Saturated incidence rates	0.005 day$^{-1}$	Assumed
$\Lambda _h^{\sigma }$	Human recruitment rate	$\frac{4{,}000{,}000}{78\times 365}$ day$^{-1}$	^35^
$\mu _h^{\sigma }$	Natural death rates	$\frac{1}{78 \times 365}$ day$^{-1}$	^35^
$\delta _{h1}^{\sigma }$	Immunity loss from individuals recovered from strain 1	0.026 day$^{-1}$	^36^
$\delta _{h2}^{\sigma }$	Immunity loss from individuals recovered from strain 2	0.026 day$^{-1}$	^36^
$\delta _{h12}^{\sigma }$	Immunity loss from individuals recovered from co-infection	0.026 day$^{-1}$	^36^
$\phi _1^{\sigma }$	Vaccine efficacy against Strain 1	[0.81,0.88] day$^{-1}$	^37^
$\phi _2^{\sigma }$	Vaccine efficacy against Strain 2	[0.81,0.88] day$^{-1}$	^37^
$\xi _{e1}^{\sigma },\xi _{e2}^{\sigma },\xi _{e12}^{\sigma }$	Progression rates from exposed to asymptomatic	0.1 day$^{-1}$	^38^
$\xi _{a1}^{\sigma },\xi _{a2}^{\sigma },\xi _{a12}^{\sigma }$	Progression rates from asymptomatic to symptomatic	0.1 day$^{-1}$	^38^
$\zeta _{a12}^{\sigma }$	Recovery rates from Asymptomatic individuals of co-infection	[0.11,0.15] day$^{-1}$	^37^
$\zeta _{i1}^{\sigma },\zeta _{i2}^{\sigma },\zeta _{i12}^{\sigma }$	Recovery rates from Symptomatic individuals	[0.11,0.15] day$^{-1}$	^37^
$\zeta _{h1}^{\sigma },\zeta _{h2}^{\sigma },\zeta _{h12}^{\sigma }$	Recovery rates from Quarantine individuals	[0.11,0.15] day$^{-1}$	^37^
$\theta _{a12}^{\sigma }$	Mortality rates for Asymptomatic Individuals of co-infection	0.001 day$^{-1}$	^37^
$\theta _{i1}^{\sigma },\theta _{i2}^{\sigma },\theta _{i12}^{\sigma }$	Mortality rates for Symptomatic individuals	0.001 day$^{-1}$	^37^
$\theta _{h1}^{\sigma },\theta _{h2}^{\sigma },\theta _{h12}^{\sigma }$	Mortality rates for Quarantine individuals	0.001 day$^{-1}$	^37^
$\eta _{i1}^{\sigma },\eta _{i2}^{\sigma },\eta _{i12}^{\sigma }$	Rates for which people are quarantine	0.05 day$^{-1}$	Assumed
$\Lambda _v^{\sigma }$	Mosquitos recruitment rate	20, 000 day$^{-1}$	Assumed
$\beta _{v1}^{\sigma }$	Transmission rate from human to vectors for dengue strain-1	[0.60, 0.75] day$^{-1}$	^37^
$\beta _{v2}^{\sigma }$	Transmission rate from human to vectors for dengue strain-2	[0.60, 0.75] day$^{-1}$	^37^
$\mu _v^{\sigma }$	Rate of removal of vectors	$(\frac{1}{21} - \frac{1}{7})$ day$^{-1}$	^37^
$\gamma _1^{\sigma },\gamma _2^{\sigma },\gamma _3^{\sigma },\gamma _4^{\sigma }$	Saturated incidence rates for vectors	0.0005	Assumed
$\omega _{v1}^{\sigma }$	Progression rates for vectors from Expose to infected with strain 1	0.1 day$^{-1}$	^38^
$\omega _{v2}^{\sigma }$	Progression rates for vectors from Expose to infected with strain 2	0.1 day$^{-1}$	^38^
$\rho _{v1}^{\sigma }$	Mortality of infected vectors with strain 1	negligible day$^{-1}$	^38^
$\rho _{v2}^{\sigma }$	Mortality of infected vectors with strain 2	negligible day$^{-1}$	^38^

4 $$\begin{aligned} \begin{aligned} { }^{C} {\mathcal {D}}_{0^{+}}^{\sigma } S_{h}(t)&=\Lambda _h^{\sigma } - \frac{\beta _{h1}^{\sigma } I_{v1}}{1+\alpha _1^{\sigma } I_{v1}} S_{h} - \frac{\beta _{h2}^{\sigma } I_{v2}}{1+\alpha _2^{\sigma } I_{v2}} S_{h} - (\mu _{h}^{\sigma } + \psi ^{\sigma }) S_{h} + \delta _{h1}^{\sigma } R_{h1} + \delta _{h2}^{\sigma } R_{h2} + \delta _{h12}^{\sigma } R_{h12}, \\ { }^{C} {\mathcal {D}}_{0^{+}}^{\sigma } V_{h}(t)&=\psi ^{\sigma } S_{h} - (1-\phi _1^{\sigma }) \frac{\beta _{h1}^{\sigma } I_{v1}}{1+\alpha _1^{\sigma } I_{v1}} V_{h} - (1-\phi _2^{\sigma }) \frac{\beta _{h2}^{\sigma } I_{v2}}{1+\alpha _2^{\sigma } I_{v2}} V_{h} - \mu _{h}^{\sigma } V_{h}, \\ { }^{C} {\mathcal {D}}_{0^{+}}^{\sigma } E_{h1}(t)&=\frac{\beta _{h1}^{\sigma } I_{v1}}{1+\alpha _1^{\sigma } I_{v1}} [S_{h} + (1-\phi _1^{\sigma })V_{h}] - (\mu _{h}^{\sigma } + \xi _{e1}^{\sigma }) E_{h1} - \frac{\beta _{h2}^{\sigma } I_{v2}}{1+\alpha _2^{\sigma } I_{v2}} E_{h1}, \\ { }^{C} {\mathcal {D}}_{0^{+}}^{\sigma } E_{h2}(t)&=\frac{\beta _{h2}^{\sigma } I_{v2}}{1+\alpha _2^{\sigma } I_{v2}} [S_{h} + (1-\phi _2^{\sigma })V_{h}] - (\mu _{h}^{\sigma } + \xi _{e2}^{\sigma }) E_{h2} - \frac{\beta _{h1}^{\sigma } I_{v1}}{1+\alpha _1^{\sigma } I_{v1}} E_{h2}, \\ { }^{C} {\mathcal {D}}_{0^{+}}^{\sigma } E_{h12}(t)&=\frac{\beta _{h2}^{\sigma } I_{v2}}{1+\alpha _2^{\sigma } I_{v2}} E_{h1} + \frac{\beta _{h1}^{\sigma } I_{v1}}{1+\alpha _1^{\sigma } I_{v1}} E_{h2}- (\mu _{h}^{\sigma } + \xi _{e12}^{\sigma }) E_{h12}, \\ { }^{C} {\mathcal {D}}_{0^{+}}^{\sigma } A_{h1}(t)&=\xi _{e1}^{\sigma } E_{h1} - (\mu _{h}^{\sigma } + \xi _{a1}^{\sigma } + \zeta _{a1}^{\sigma } + \theta _{a1}^{\sigma }) A_{h1} - \frac{\beta _{h2}^{\sigma } I_{v2}}{1+\alpha _2^{\sigma } I_{v2}} A_{h1}, \\ { }^{C} {\mathcal {D}}_{0^{+}}^{\sigma } A_{h2}(t)&=\xi _{e2}^{\sigma } E_{h2} - (\mu _{h}^{\sigma } + \xi _{a2}^{\sigma } + \zeta _{a2}^{\sigma } + \theta _{a2}^{\sigma }) A_{h2} - \frac{\beta _{h1}^{\sigma } I_{v1}}{1+\alpha _1^{\sigma } I_{v1}} A_{h2}, \\ { }^{C} {\mathcal {D}}_{0^{+}}^{\sigma } A_{h12}(t)&=\xi _{e12}^{\sigma } E_{h12} - (\mu _{h}^{\sigma } + \xi _{a12}^{\sigma } + \zeta _{a12}^{\sigma } + \theta _{a12}^{\sigma }) A_{h12} + \frac{\beta _{h2}^{\sigma } I_{v2}}{1+\alpha _2^{\sigma } I_{v2}} A_{h1} + \frac{\beta _{h1}^{\sigma } I_{v1}}{1+\alpha _1^{\sigma } I_{v1}} A_{h2}, \\ { }^{C} {\mathcal {D}}_{0^{+}}^{\sigma } I_{h1}(t)&=\xi _{a1}^{\sigma } A_{h1} - (\mu _{h}^{\sigma } + \zeta _{i1}^{\sigma } + \theta _{i1}^{\sigma } + \eta _{i1}^{\sigma }) I_{h1} - \frac{\beta _{h2}^{\sigma } I_{v2}}{1+\alpha _2^{\sigma } I_{v2}} I_{h1}, \\ { }^{C} {\mathcal {D}}_{0^{+}}^{\sigma } I_{h2}(t)&=\xi _{a2}^{\sigma } A_{h2} - (\mu _{h}^{\sigma } + \zeta _{i2}^{\sigma } + \theta _{i2}^{\sigma } + \eta _{i2}^{\sigma }) I_{h2} - \frac{\beta _{h1}^{\sigma } I_{v1}}{1+\alpha _1^{\sigma } I_{v1}} I_{h2}, \\ { }^{C} {\mathcal {D}}_{0^{+}}^{\sigma } I_{h12}(t)&=\xi _{a12}^{\sigma } A_{h12} - (\mu _{h}^{\sigma } + \zeta _{i12}^{\sigma } + \theta _{i12}^{\sigma } + \eta _{i12}^{\sigma }) I_{h12} + \frac{\beta _{h2}^{\sigma } I_{v2}}{1+\alpha _2^{\sigma } I_{v2}} I_{h1} + \frac{\beta _{h1}^{\sigma } I_{v1}}{1+\alpha _1^{\sigma } I_{v1}} I_{h2}, \\ { }^{C} {\mathcal {D}}_{0^{+}}^{\sigma } Q_{h1}(t)&=\eta _{i1}^{\sigma } I_{h1} - (\mu _{h}^{\sigma } + \zeta _{h1}^{\sigma } + \theta _{h1}^{\sigma }) Q_{h1}, \\ { }^{C} {\mathcal {D}}_{0^{+}}^{\sigma } Q_{h2}(t)&=\eta _{i2}^{\sigma } I_{h2} - (\mu _{h}^{\sigma } + \zeta _{h2}^{\sigma } + \theta _{h2}^{\sigma }) Q_{h2}, \\ { }^{C} {\mathcal {D}}_{0^{+}}^{\sigma } Q_{h12}(t)&=\eta _{i12}^{\sigma } I_{h12} - (\mu _{h}^{\sigma } + \zeta _{h12}^{\sigma } + \theta _{h12}^{\sigma }) Q_{h12},\\ { }^{C} {\mathcal {D}}_{0^{+}}^{\sigma } R_{h1}(t)&=\zeta _{a1}^{\sigma } A_{h1} + \zeta _{i1}^{\sigma } I_{h1} + \zeta _{h1}^{\sigma } Q_{h1} - (\mu _{h}^{\sigma } + \delta _{h1}^{\sigma }) R_{h1}, \\ { }^{C} {\mathcal {D}}_{0^{+}}^{\sigma } R_{h2}(t)&=\zeta _{a2}^{\sigma } A_{h2} + \zeta _{i2}^{\sigma } I_{h2} + \zeta _{h2}^{\sigma } Q_{h2} - (\mu _{h}^{\sigma } + \delta _{h2}^{\sigma }) R_{h2}, \\ { }^{C} {\mathcal {D}}_{0^{+}}^{\sigma } R_{h12}(t)&=\zeta _{a12}^{\sigma } A_{h12} + \zeta _{i12}^{\sigma } I_{h12} + \zeta _{h12}^{\sigma } Q_{h12} - (\mu _{h}^{\sigma } + \delta _{h12}^{\sigma }) R_{h12}, \\ { }^{C} {\mathcal {D}}_{0^{+}}^{\sigma } S_{v}(t)&= \Lambda _v^{\sigma } - \frac{\beta _{v1}^{\sigma }(A_{h1} + I_{h1} + A_{h12} + I_{h12})}{1+\gamma _1^{\sigma } A_{h1} + \gamma _2^{\sigma } I_{h1} + \gamma _3^{\sigma } A_{h12} + \gamma _4^{\sigma } I_{h12}} S_{v} - \frac{\beta _{v2}^{\sigma }(A_{h2} + I_{h2} + A_{h12} + I_{h12})}{1+\gamma _1^{\sigma } A_{h2} + \gamma _2^{\sigma } I_{h2} + \gamma _3^{\sigma } A_{h12} + \gamma _4^{\sigma } I_{h12}} S_{v} - \mu _{v}^{\sigma } S_{v}, \\ { }^{C} {\mathcal {D}}_{0^{+}}^{\sigma } E_{v1}(t)&= \frac{\beta _{v1}^{\sigma }(A_{h1} + I_{h1} + A_{h12} + I_{h12})}{1+\gamma _1^{\sigma } A_{h1} + \gamma _2^{\sigma } I_{h1} + \gamma _3^{\sigma } A_{h12} + \gamma _4^{\sigma } I_{h12}} S_{v} - (\mu _{v}^{\sigma } + \omega _{v1}^{\sigma }) E_{v1}, \\ { }^{C} {\mathcal {D}}_{0^{+}}^{\sigma } E_{v2}(t)&= \frac{\beta _{v2}^{\sigma }(A_{h2} + I_{h2} + A_{h12} + I_{h12})}{1+\gamma _1^{\sigma } A_{h2} + \gamma _2^{\sigma } I_{h2} + \gamma _3^{\sigma } A_{h12} + \gamma _4^{\sigma } I_{h12}} S_{v} - (\mu _{v}^{\sigma } + \omega _{v2}^{\sigma }) E_{v2},\\ { }^{C} {\mathcal {D}}_{0^{+}}^{\sigma } I_{v1}(t)&= \omega _{v1}^{\sigma } E _{v1}- (\mu _{v}^{\sigma } + \rho _{v1}^{\sigma }) I_{v1},\\ { }^{C} {\mathcal {D}}_{0^{+}}^{\sigma } I_{v2}(t)&= \omega _{v2}^{\sigma } E _{v2}- (\mu _{v}^{\sigma } + \rho _{v2}^{\sigma }) I_{v2}. \\ \end{aligned} \end{aligned}$$

The model can be written in compact form as:

5 $$\begin{aligned} {\left\{ \begin{array}{ll} ^{C}D^{\sigma }_{t}{\varPhi }(t) &{} ={\mathcal {K}}(t,{\varPhi }(t)), \\ {\varPhi }(0) &{} =\varPhi _0, \end{array}\right. } \end{aligned}$$

**Figure 1:**
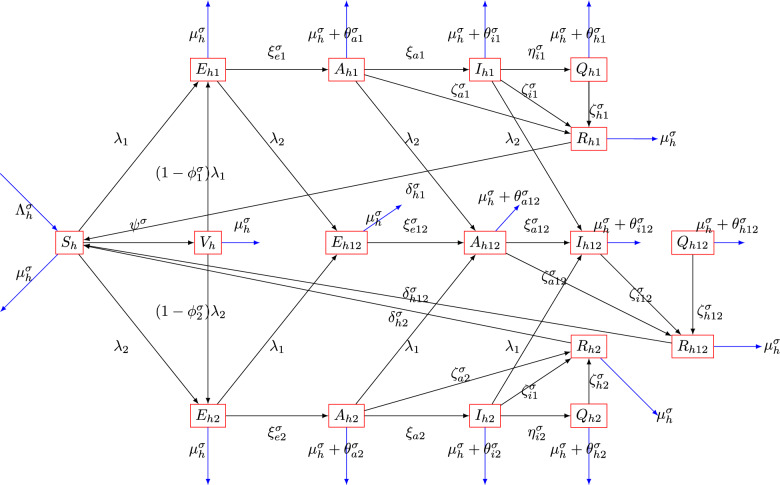
Schematic diagram of the model (4) where $\lambda _{1}=\frac{\beta _{h1}^{\sigma }I_{v1}}{1+\alpha _{1}^{\sigma }I_{v1}} ~and~\lambda _{2}=\frac{\beta _{h2}^{\sigma }I_{v2}}{1+\alpha _{2}^{\sigma }I_{v2}}$.

The diagram of the model (4) is given in the Fig. 1.

## Analysis of the sub-models

The completel model has 22 equations, which is quite complex to analyze qualitatively. We will therefore, consider the strain 1 and strain 2 sub-models for local and global stability analyses. The sub-models analyses results will help to examine the stability of the complete model.

### Analysis of the strain 1 only sub-model

The strain 1-only sub-model is obtained by setting the classes $E_{h2} = E_{h12}=A_{h2} =A_{h12}=I_{h2} =I_{h12}=Q_{h2} =Q_{h12}=R_{h2} =R_{h12} = E_{v2}=I_{v2}$ equal to 0. The model is given by:

6 $$\begin{aligned} \begin{aligned} { }^{C} {\mathcal {D}}_{0^{+}}^{\sigma } S_{h}(t)&=\Lambda _h^{\sigma } - \frac{\beta _{h1}^{\sigma } I_{v1}}{1+\alpha _1^{\sigma } I_{v1}} S_{h} - (\mu _{h}^{\sigma } + \psi ^{\sigma }) S_{h} + \delta _{h1}^{\sigma } R_{h1}, \\ { }^{C} {\mathcal {D}}_{0^{+}}^{\sigma } V_{h}(t)&=\psi ^{\sigma } S_{h} - (1-\phi _1^{\sigma }) \frac{\beta _{h1}^{\sigma } I_{v1}}{1+\alpha _1^{\sigma } I_{v1}} V_{h} - \mu _{h}^{\sigma } V_{h}, \\ { }^{C} {\mathcal {D}}_{0^{+}}^{\sigma } E_{h1}(t)&=\frac{\beta _{h1}^{\sigma } I_{v1}}{1+\alpha _1^{\sigma } I_{v1}} [S_{h} + (1-\phi _1^{\sigma })V_{h}] - (\mu _{h}^{\sigma } + \xi _{e1}^{\sigma }) E_{h1}, \\ { }^{C} {\mathcal {D}}_{0^{+}}^{\sigma } A_{h1}(t)&=\xi _{e1}^{\sigma } E_{h1} - (\mu _{h}^{\sigma } + \xi _{a1}^{\sigma } + \zeta _{a1}^{\sigma } + \theta _{a1}^{\sigma }) A_{h1}, \\ { }^{C} {\mathcal {D}}_{0^{+}}^{\sigma } I_{h1}(t)&=\xi _{a1}^{\sigma } A_{h1} - (\mu _{h}^{\sigma } + \zeta _{i1}^{\sigma } + \theta _{i1}^{\sigma } + \eta _{i1}^{\sigma }) I_{h1}, \\ { }^{C} {\mathcal {D}}_{0^{+}}^{\sigma } Q_{h1}(t)&=\eta _{i1}^{\sigma } I_{h1} - (\mu _{h}^{\sigma } + \zeta _{h1}^{\sigma } + \theta _{h1}^{\sigma }) Q_{h1}, \\ { }^{C} {\mathcal {D}}_{0^{+}}^{\sigma } R_{h1}(t)&=\zeta _{a1}^{\sigma } A_{h1} + \zeta _{i1}^{\sigma } I_{h1} + \zeta _{h1}^{\sigma } Q_{h1} - (\mu _{h}^{\sigma } + \delta _{h1}^{\sigma }) R_{h1}, \\ { }^{C} {\mathcal {D}}_{0^{+}}^{\sigma } S_{v}(t)&= \Lambda _v^{\sigma } - \frac{\beta _{v1}^{\sigma }(A_{h1} + I_{h1})}{1+\gamma _1^{\sigma } A_{h1} + \gamma _2^{\sigma } I_{h1}} S_{v} - \mu _{v}^{\sigma } S_{v}, \\ { }^{C} {\mathcal {D}}_{0^{+}}^{\sigma } E_{v1}(t)&= \frac{\beta _{v1}^{\sigma }(A_{h1} + I_{h1})}{1+\gamma _1^{\sigma } A_{h1} + \gamma _2^{\sigma } I_{h1}} S_{v} - (\mu _{v}^{\sigma } + \omega _{v1}^{\sigma }) E_{v1}, \\ { }^{C} {\mathcal {D}}_{0^{+}}^{\sigma } I_{v1}(t)&= \omega _{v1}^{\sigma } E _{v1}- (\mu _{v}^{\sigma } + \rho _{v1}^{\sigma }) I_{v1}.\\ \end{aligned} \end{aligned}$$

The strain-1 only infection-free equilibrium is given by:

$$\begin{aligned} {\mathcal {D}}_{01} = \left( \frac{\Lambda _h^{\sigma }}{\mu _h^{\sigma }+\psi ^{\sigma }},\frac{\psi ^{\sigma }}{\mu _h^{\sigma }}\bigg (\frac{\Lambda _h^{\sigma }}{\mu _h^{\sigma }+\psi ^{\sigma }}\bigg ), 0, 0, 0, 0, 0, 0, 0, \frac{\Lambda _v}{\mu _v}, 0\right) . \end{aligned}$$

The matrix of new infection is given as:

7 $$\begin{aligned} F=\begin{bmatrix} 0&{}0&{}0&{}0&{}0&{}\beta _{h1}^{\sigma }A_1^*\\ 0&{}0&{}0&{}0&{}0&{}0\\ 0&{}0&{}0&{}0&{}0&{}0\\ 0&{}0&{}0&{}0&{}0&{}0\\ 0&{}\beta _{v1}^{\sigma }A_{2}^{*}&{}\beta _{v1}^{\sigma }A_{2}^{*}&{}0&{}0&{}0\\ 0&{}0&{}0&{}0&{}0&{}0\\ \end{bmatrix}, \end{aligned}$$

where $$A_{1}^{*}=(S_{h}^{*}+(1-\phi _{1}^{\sigma })V_{h}^{*}), A_{2}^{*}=S_{v}^{*}.$$

The matrix for transfer of infection by all other means is given as:

8 $$\begin{aligned} V=\begin{bmatrix} K_{1}&{}0&{}0&{}0&{}0&{}0\\ -\xi _{e1}^{\sigma }&{}K_{2}&{}0&{}0&{}0&{}0\\ 0&{}-\xi _{a1}^{\sigma }&{}K_{3}&{}0&{}0&{}0\\ 0&{}0&{}-\eta _{i1}^{\sigma }&{}K_{4}&{}0&{}0\\ 0&{}0&{}0&{}0&{}K_{5}&{}0\\ 0&{}0&{}0&{}0&{}-\omega _{v1}^{\sigma }&{}K_{6}\\ \end{bmatrix}, \end{aligned}$$

where $$K_{1}=(\mu _{h}^{\sigma } + \xi _{e1}^{\sigma }),K_{2}=(\mu _{h}^{\sigma } + \xi _{a1}^{\sigma } + \zeta _{a1}^{\sigma } + \theta _{a1}^{\sigma }),K_{3}=(\mu _{h}^{\sigma } + \zeta _{i1}^{\sigma } + \theta _{i1}^{\sigma } + \eta _{i1}^{\sigma })$$

$$K_{4}=(\mu _{h}^{\sigma } + \zeta _{h1}^{\sigma } + \theta _{h1}^{\sigma }),K_{5}=(\mu _{v}^{\sigma } + \omega _{v1}^{\sigma }),K_{6}=(\mu _{v}^{\sigma } + \rho _{v1}^{\sigma }).$$

The reproduction number ${\mathcal {R}}_{01}$ for strain-1 is given as:

$$\begin{aligned} {\mathcal {R}}_{01}=\sqrt{\frac{\beta _{h1}^{\sigma }\beta _{v1}^{\sigma }A_{1}^{*}A_{2} ^{*}\xi _{e1}^{\sigma }\omega _{v1}^{\sigma }(K_{3}+\xi _{a1}^{\sigma })}{K_{1}K_{2}K_{3}K_{5}K_{6}}}. \end{aligned}$$

This can be written as:

$$\begin{aligned} {\mathcal {R}}_{01}=\sqrt{{\mathcal {R}}_{1H}{\mathcal {R}}_{1V}}. \end{aligned}$$

where $${\mathcal {R}}_{1V}=\frac{\beta _{h1}^{\sigma }A_{1}^{*}\omega _{v1}^{\sigma }}{K_{5}K_{6}}$$ and $${\mathcal {R}}_{1H}=\frac{\beta _{v1}^{\sigma }A_{2}^{*}\xi _{e1}^{\sigma }}{K_{1}K_{2}} +\frac{\beta _{v1}^{\sigma }A_{2}^{*}\xi _{e1}^{\sigma }\xi _{a1}^{\sigma }}{K_{1}K_{2}K_{3}}$$.

The term ${\mathcal {R}}_{1V}$ is the average number of new dengue strain-1 infections generated by one infected vector who progresses through the stages ${\mathcal {E}}_{v1} \rightarrow {\mathcal {I}}_{v1},$ in the susceptible human population.The term $\frac{\omega _{v1}^{\sigma }}{K_{5}}$ accounts for the average duration of infection in vectors in the exposed class while $\frac{1}{K_{6}}$ accounts for the average duration of infection in vectors in the infected stage. The term is product of the transmission rate of susceptible human by dengue infected mosquitoes $(\beta _{h1}^{\sigma })$ and the mean duration of infection in the mosquitoes $\frac{\omega _{v1}^{\sigma }}{K_{5}K_{6}}.$

The term ${\mathcal {R}}_{1H}$ is the average number of new dengue strain-1 infections in vectors generated by one infected individual who progresses through the stages ${\mathcal {E}}_{h1} \rightarrow {\mathcal {A}}_{h1}$ or ${\mathcal {E}}_{h1} \rightarrow {\mathcal {A}}_{h1} \rightarrow {\mathcal {I}}_{h1}$. The term $\frac{\beta _{v1}^{\sigma }A_{2}^{*}\xi _{e1}^{\sigma }\xi _{a1}^{\sigma }}{K_{1}K_{2}K_{3}}$ is the product of transmission rate in mosquitoes by a typical infected human $\beta _{v1}^{\sigma }$ and the mean duration of infection in human $\frac{\xi _{e1}^{\sigma }\xi _{a1}^{\sigma }}{K_{1}K_{2}K_{3}}.$ The explanation of the the term $\frac{\xi _{e1}^{\sigma }\xi _{a1}^{\sigma }}{K_{1}K_{2}K_{3}}.$ is given as

- $\frac{\xi _{e1}^{\sigma }}{K_{1}}$ accounts for the duration of infection in human in the exposed class.
- $\frac{\xi _{a1}^{\sigma }}{K_{2}}$ accounts for the duration of infection in human in the asymptomatic class.
- $\frac{1}{K_{3}}$ accounts for the duration of infection in human in the infected (symptomatic) class.

Consider the Lyapunov function:

9 $$\begin{aligned} \begin{aligned} {\mathcal {L}}_{1}&=\bigg (\frac{A_{2}^{*}\beta _{v1}^{\sigma }\omega _{v1}^{\sigma }\xi _{e1} ^{\sigma }}{K_{1}K_{2}K_{5}K_{6}}+\frac{A_{2}^{*}\beta _{v1}^{\sigma }\omega _{v1}^{\sigma } \xi _{e1}^{\sigma }\xi _{a1}^{\sigma }}{K_{1}K_{2}K_{3}K_{5}K_{6}}\bigg )E_{h1}+\bigg (\frac{A_{2} ^{*}\beta _{v1}^{\sigma }\omega _{v1}^{\sigma }}{K_{2}K_{5}K_{6}}+\frac{A_{2}^{*}\beta _{v1}^{\sigma } \omega _{v1}^{\sigma }\xi _{a1}^{\sigma }}{K_{2}K_{3}K_{5}K_{6}}\bigg )A_{h1}\\&\quad +\bigg (\frac{A_{2}^{*}\beta _{v1}^{\sigma }\omega _{v1}^{\sigma }}{K_{3}K_{5}K_{6}}\bigg )I_{h1} +\bigg (\frac{\omega _{v1}^{\sigma }{\mathcal {R}}_{01}}{K_{5}K_{6}}\bigg )E_{v1} +\bigg (\frac{{\mathcal {R}}_{01}}{K_{6}}\bigg )I_{v1}. \end{aligned} \end{aligned}$$

The time derivative of fractional order $\sigma$ is given by:

10 $$\begin{aligned} \begin{aligned} ^{C}D^{\sigma }_{0^{+}}{\mathcal {L}}_{1}&=\bigg (\frac{A_{2}^{*}\beta _{v1}^{\sigma } \omega _{v1}^{\sigma }\xi _{e1}^{\sigma }}{K_{1}K_{2}K_{5}K_{6}}+\frac{A_{2}^{*}\beta _{v1} ^{\sigma }\omega _{v1}^{\sigma }\xi _{e1}^{\sigma }\xi _{a1}^{\sigma }}{K_{1}K_{2}K_{3}K_{5}K_{6}} \bigg )~^{C}D^{\sigma }_{0^{+}}E_{h1}+\bigg (\frac{A_{2}^{*}\beta _{v1}^{\sigma }\omega _{v1} ^{\sigma }}{K_{2}K_{5}K_{6}}+\frac{A_{2}^{*}\beta _{v1}^{\sigma }\omega _{v1}^{\sigma }\xi _{a1} ^{\sigma }}{K_{2}K_{3}K_{5}K_{6}}\bigg )~^{C}D^{\sigma }_{0^{+}}A_{h1}\\&\quad +\bigg (\frac{A_{2}^{*}\beta _{v1}^{\sigma }\omega _{v1}^{\sigma }}{K_{3}K_{5}K_{6}}\bigg ) ~^{C}D^{\sigma }_{0^{+}}I_{h1}+\bigg (\frac{\omega _{v1}^{\sigma }{\mathcal {R}}_{01}}{K_{5} K_{6}}\bigg )~^{C}D^{\sigma }_{0^{+}}E_{v1}+\bigg (\frac{{\mathcal {R}}_{01}}{K_{6}}\bigg )~ ^{C}D^{\sigma }_{0^{+}}I_{v1}. \end{aligned} \end{aligned}$$

Substituting the values of the fractional derivatives for each compartments gives:

11 $$\begin{aligned} \begin{aligned} ^{C}D^{\sigma }_{0^{+}}{\mathcal {L}}_{1}&=\bigg (\frac{A_{2}^{*}\beta _{v1}^{\sigma } \omega _{v1}^{\sigma }\xi _{e1}^{\sigma }}{K_{1}K_{2}K_{5}K_{6}}+\frac{A_{2}^{*}\beta _{v1} ^{\sigma }\omega _{v1}^{\sigma }\xi _{e1}^{\sigma }\xi _{a1}^{\sigma }}{K_{1}K_{2}K_{3}K_{5}K_{6}} \bigg )\bigg (\frac{\beta _{h1}^{\sigma } I_{v1}}{1+\alpha _1^{\sigma } I_{v1}} [S_{h} + (1-\phi _1^{\sigma })V_{h}] - K_{1} E_{h1}\bigg )\\&\quad +\bigg (\frac{A_{2}^{*}\beta _{v1}^{\sigma }\omega _{v1}^{\sigma }}{K_{2}K_{5}K_{6}} +\frac{A_{2}^{*}\beta _{v1}^{\sigma }\omega _{v1}^{\sigma }\xi _{a1}^{\sigma }}{K_{2}K_{3}K_{5} K_{6}}\bigg )\bigg (\xi _{e1}^{\sigma } E_{h1} - K_{2} A_{h1}\bigg )+\bigg (\frac{A_{2}^{*}\beta _{v1}^{\sigma }\omega _{v1}^{\sigma }}{K_{3}K_{5}K_{6}} \bigg )\bigg (\xi _{a1}^{\sigma } A_{h1} - K_{3} I_{h1}\bigg )\\&\quad +\bigg (\frac{\omega _{v1}^{\sigma }{\mathcal {R}}_{01}}{K_{5}K_{6}}\bigg ) \bigg (\frac{\beta _{v1}^{\sigma }(A_{h1} + I_{h1})}{1+\gamma _1^{\sigma } A_{h1} + \gamma _2^{\sigma } I_{h1}} S_{v} - K_{5} E_{v1}\bigg )+\bigg (\frac{{\mathcal {R}}_{01}}{K_{6}}\bigg )\bigg (\omega _{v1}^{\sigma } E _{v1}- K_{6} I_{v1}\bigg ), \end{aligned} \end{aligned}$$

which can also be written thus:

12 $$\begin{aligned} \begin{aligned} ^{C}D^{\sigma }_{0^{+}}{\mathcal {L}}_{1}&\le \bigg (\frac{A_{2}^{*}\beta _{v1}^{\sigma } \omega _{v1}^{\sigma }\xi _{e1}^{\sigma }}{K_{1}K_{2}K_{5}K_{6}}+\frac{A_{2}^{*}\beta _{v1} ^{\sigma }\omega _{v1}^{\sigma }\xi _{e1}^{\sigma }\xi _{a1}^{\sigma }}{K_{1}K_{2}K_{3}K_{5}K_{6}} \bigg )\bigg (\beta _{h1}^{\sigma } I_{v1}(A_{1}^{*}) - K_{1} E_{h1}\bigg )\\&\quad +\bigg (\frac{A_{2}^{*}\beta _{v1}^{\sigma }\omega _{v1}^{\sigma }}{K_{2}K_{5}K_{6}} +\frac{A_{2}^{*}\beta _{v1}^{\sigma }\omega _{v1}^{\sigma }\xi _{a1}^{\sigma }}{K_{2}K_{3}K_{5}K_{6}}\bigg ) \bigg (\xi _{e1}^{\sigma } E_{h1} - K_{2} A_{h1}\bigg )+\bigg (\frac{A_{2}^{*}\beta _{v1}^{\sigma }\omega _{v1} ^{\sigma }}{K_{3}K_{5}K_{6}}\bigg )\bigg (\xi _{a1}^{\sigma } A_{h1} - K_{3} I_{h1}\bigg )\\&\quad +\bigg (\frac{\omega _{v1}^{\sigma }{\mathcal {R}}_{01}}{K_{5}K_{6}}\bigg )\bigg (\beta _{v1}^{\sigma } (A_{h1} + I_{h1})A_{2}^{*} - K_{5} E_{v1}\bigg )+\bigg (\frac{{\mathcal {R}}_{01}}{K_{6}}\bigg ) \bigg (\omega _{v1}^{\sigma } E _{v1}- K_{6} I_{v1}\bigg ),\\ \end{aligned} \end{aligned}$$

which on simplification gives:

13 $$\begin{aligned} ^{C}D^{\sigma }_{0^{+}}{\mathcal {L}}_{1}\le {\mathcal {R}}_{01}\bigg ({\mathcal {R}}_{01}-1\bigg )I_{v1}+\frac{\omega _{v1}^{\sigma } \beta _{v1}^{\sigma }A_{2}^{*}}{K_{5}K_{6}}\bigg ({\mathcal {R}}_{01}-1\bigg )A_{h1} +\frac{\omega _{v1}^{\sigma }\beta _{v1}^{\sigma }A_{2}^{*}}{K_{5}K_{6}}\bigg ({\mathcal {R}}_{01}-1\bigg )I_{h1}. \end{aligned}$$

Clearly ${\mathcal {R}}_{\tiny \textsc {01}}\le 1$ is satisfied since the above inequality with positive parameters shows that $D_t^\alpha {\mathcal {L}}_1$ is negative semi definite. It follows from the results in^39,40^ that the disease free equilibrium is globally asymptotically stable if ${\mathcal {R}}_{\tiny \textsc {01}}\le 1$ and unstable if ${\mathcal {R}}_{\tiny \textsc {01}}>1$.

### Local stability for sub-model of strain-1

The stability of system (6) in the neighborhood of the DFE is analyzed by Jacobian of system (6) evaluated at DFE ${\mathcal {D}}_{01}$, which is given as:

14 $$\begin{aligned} \begin{bmatrix} -K_{0}&{}0&{}0&{}0&{}0&{}0&{}\delta _{h1}^{\sigma }&{}0&{}0&{}-\beta _{h1}^{\sigma }S_{h}^{*}\\ \psi ^{\sigma }_{h}&{}-\mu _h^{\sigma }&{}0&{}0&{}0&{}0&{}0&{}0&{}0&{}-(1-\phi _1^{\sigma })\beta _{h1}^{\sigma }V_{h}^*\\ 0&{}0&{}-K_{1}&{}0&{}0&{}0&{}0&{}0&{}0&{}\beta _{h1}^{\sigma }A_{1}^{*}\\ 0&{}0&{}\xi _{e1}^{\sigma }&{}-K_{2}&{}0&{}0&{}0&{}0&{}0&{}0\\ 0&{}0&{}0&{}\xi _{a1}^{\sigma }&{}-K_{3}&{}0&{}0&{}0&{}0&{}0\\ 0&{}0&{}0&{}0&{}\eta _{i1}^{\sigma }&{}-K_{4}&{}0&{}0&{}0&{}0\\ 0&{}0&{}0&{}\zeta _{a1}^{\sigma }&{}\zeta _{i1}^{\sigma }&{}\zeta _{h1}^{\sigma }&{}-K_{13}&{}0&{}0&{}0\\ 0&{}0&{}0&{}-\beta _{v1}^{\sigma }A_{2}^{*}&{}-\beta _{v1}^{\sigma }A_{2}^{*}&{}0&{}0&{}-\mu _v^{\sigma }&{}0&{}0\\ 0&{}0&{}0&{}\beta _{v1}^{\sigma }A_{2}^{*}&{}\beta _{v1}^{\sigma }A_{2}^{*}&{}0&{}0&{}0&{}-K_{5}&{}0\\ 0&{}0&{}0&{}0&{}0&{}0&{}0&{}0&{}\omega _{v1}^{\sigma }&{}-K_{6}\\ \end{bmatrix}, \end{aligned}$$

where

$$\begin{aligned} K_{0}=\mu _{h}^{\sigma }+\psi ^{\sigma },K_{13}=\mu _{h}^{\sigma }+\delta _{h1}^{\sigma } \end{aligned}$$

The “characteristic polynomial” is given by:

15 $$\begin{aligned}&(\lambda +K_{0})(\lambda +K_{4})(\lambda +K_{13})(\lambda +\mu _h^{\sigma })(\lambda +\mu _v^{\sigma }) \bigg ((\lambda +K_{1})(\lambda +K_{2})(\lambda \\ {}&+K_{3})(\lambda +K_{5})(\lambda +K_{6})-A_{1}^{*} A_{2}^{*}\beta _{h1}^{\sigma }\beta _{v1}^{\sigma }\xi _{e1}^{\sigma }(\lambda +K_{3}+\xi _{a1}^{\sigma }) \omega _{v1}^{\sigma }\bigg )=0. \end{aligned}$$

This can be written as:

16 $$\begin{aligned}{} & {} (\lambda +K_{0})(\lambda +K_{4})(\lambda +K_{13})(\lambda +\mu _h^{\sigma })(\lambda +\mu _v^{\sigma }) \bigg (\lambda ^{5}+a_{11}\lambda ^{4}+a_{12}\lambda ^{3}\nonumber \\{} & {} \quad +a_{13}\lambda ^{2}+a_{14}\lambda +K_{1}K_{2} K_{3}K_{5}K_{6}\bigg (1-R_{01}^{2}\bigg )\bigg )=0, \end{aligned}$$

where

17 $$\begin{aligned} \begin{aligned} a_{11}&=K_{1}+K_{2}+K_{3}+K_{5}+K_{6}\\ a_{12}&=K_{1}K_{2}+K_{1}K_{3}+K_{2}K_{3}+K_{1}K_{5}+K_{2}K_{5}+K_{3}K_{5}+K_{1}K_{6}+K_{2}K_{6} +K_{3}K_{6}+K_{5}K_{6}\\ a_{13}&=K_{1}K_{2}K_{3}+K_{1}K_{2}K_{5}+K_{1}K_{3}K_{5}+K_{2}K_{3}K_{5}+K_{1}K_{2}K_{6}\\&\quad +K_{1}K_{3}K_{6}+K_{2}K_{3}K_{6}+K_{1}K_{5}K_{6}+K_{2}K_{5}K_{6}+K_{3}K_{5}K_{6}\\ a_{14}&=K_{1}K_{2}K_{3}K_{5}+K_{1}K_{2}K_{3}K_{6}+K_{1}K_{2}K_{5}K_{6}+K_{1}K_{3}K_{5}K_{6} +K_{2}K_{3}K_{5}K_{6}-A_{1}^*A_{2}^*\beta _{h1}^{\sigma }\beta _{v1}^{\sigma }\xi _{e1}^{\sigma } \omega _{v1}^{\sigma }.\\ \end{aligned} \end{aligned}$$

The eigenvalues are given by:

$$\begin{aligned} \begin{aligned} \lambda _1 = - \mu _v^{\sigma }, \quad \lambda _2 = - K_{0}, \quad \lambda _3 = - K_{4}, \quad \lambda _4 = -\mu _h^{\sigma } \quad \lambda _5 = - K_{13}, \end{aligned} \end{aligned}$$

and the solution of the equations is given by

18 $$\begin{aligned} \bigg (\lambda ^{5}+a_{11}\lambda ^{4}+a_{12}\lambda ^{3}+a_{13}\lambda ^{2}+a_{14} \lambda +K_{1}K_{2}K_{3}K_{5}K_{6}\bigg (1-R_{01}^{2}\bigg )\bigg ). \end{aligned}$$

From the Routh–Hurwitz criterion, the Eq. (18) has roots with negative real parts if and only if ${\mathcal {R}}_{01}<1$. Hence, the DFE is locally asymptotically stable if ${\mathcal {R}}_{01} <1$.

Similar results can also be established for the Strain 2 only sub-model.

## Analysis of the complete model

### Invariant domain

#### Theorem 4.1

The closed set ${\mathcal {D}} = {\mathcal {D}}_h \times {\mathcal {D}}_v$, where

$$\begin{aligned}{} & {} \begin{aligned} {\mathcal {D}}_h&=\bigg .\bigg \{(S_{h}(t), V_h(t),E_{h1}(t),E_{h2}(t),E_{h12}(t),A_{h1}(t),A_{h2}(t),A_{h12}(t),I_{h1}(t),I_{h2}(t), I_{h12}(t),Q_{h1}(t),Q_{h2}(t),Q_{h12}(t), \\ {}&\quad \quad R_{h1}(t),R_{h2}(t),R_{h12}(t)) \in {\mathfrak {R}}_{+}^{17}: \\&\quad \quad S_{h}(t)+ V_h(t)+E_{h1}(t)+E_{h2}(t)+E_{h12}(t)+A_{h1}(t)+A_{h2}(t)+A_{h12}(t)+I_{h1}(t)+I_{h2}(t)+I_{h12}(t)+\\&\quad \quad Q_{h1}(t)+Q_{h2}(t)+Q_{h12}(t)+ R_{h1}(t)+R_{h2}(t)+R_{h12}(t) \le \frac{\Lambda _h^{\sigma }}{\mu _h^{\sigma }}\bigg .\bigg \},\\ \end{aligned} \\{} & {} \quad \begin{aligned} {\mathcal {D}}_v&=\bigg .\bigg \{(S_{v}(t), E_{v1}(t),E_{v2}(t),I_{v1}(t),I_{v2}(t)) \in {\mathfrak {R}}_{+}^{5}: \\&\quad \quad S_{v}(t)+ E_{v1}(t)+E_{v2}(t)+I_{v1}(t)+I_{v2}(t) \le \frac{\Lambda _v^{\sigma }}{\mu _v^{\sigma }} \bigg .\bigg \},\\ \end{aligned} \end{aligned}$$

is positively invariant in relation to the system (4).

#### Proof

Adding all the human components of the system (4), we have

19 $$\begin{aligned} \begin{aligned} ^{C}_{0} D^\omega _t N_h&= \Lambda _h^{\sigma } - \mu N_h(t) - [\theta _{a1}^{\sigma } A_{h1} +\theta _{a2}^{\sigma } A_{h2}+\theta _{a12}^{\sigma } A_{h12}+\theta _{i1}^{\sigma } I_{h1}\\&\quad +\theta _{i2}^{\sigma } I_{h2}+\theta _{i12}^{\sigma } I_{h12}+\theta _{h1}^{\sigma } Q_{h1} +\theta _{h2}^{\sigma } Q_{h2}+\theta _{h12}^{\sigma } Q_{h12}]. \end{aligned} \end{aligned}$$

From (19), we have

20 $$\begin{aligned} ^{C}_{0} D^\sigma _t N < \Lambda _h^{\sigma } - \mu _h^{\sigma } N_h. \end{aligned}$$

Applying Laplace transform on both sides of the inequality (20), we obtain that

$$\begin{aligned} s^\sigma {\mathcal {L}}\{N_h(t)\}-s^{\sigma -1}N(0)\le \frac{\Lambda _h^{\sigma }}{s}-\mu _h^{\sigma } {\mathcal {L}}\{N_h(t)\}, \end{aligned}$$

which further implies that

21 $$\begin{aligned} {\mathcal {L}}\{N_h(t)\}\le \frac{\Lambda _h^{\sigma }}{s(s^\sigma +\mu _h^{\sigma })} +N_h(0)\frac{s^{\sigma -1}}{s^\sigma +\mu _h^{\sigma }}. \end{aligned}$$

By partial fraction, the above expression reduces to

22 $$\begin{aligned} {\mathcal {L}}\{N_h(t)\}\le \frac{\Lambda _h^{\sigma }}{\mu _h^{\sigma }} \left( \frac{1}{s} \right) -\left( \frac{\Lambda _h^{\sigma }}{\mu _h^{\sigma }}-N_h(0) \right) \frac{s^{\sigma -1}}{s^\sigma +\mu _h^{\sigma }}. \end{aligned}$$

The inverse Laplace transform gives

23 $$\begin{aligned} N_h(t) \le \frac{\Lambda _h^{\sigma }}{\mu _h^{\sigma }}-\left( \frac{\Lambda _h^{\sigma }}{\mu _h^{\sigma }}-N_h(0) \right) E_\sigma \left( -\mu _h^{\sigma } t^\sigma \right) . \end{aligned}$$

Since the “Mittag-Leffler function” has asymptotic behaviour, we have

$N_h(t)\le \frac{\Lambda _h^{\sigma } }{\mu _h^{\sigma } }$ as $t\rightarrow \infty$. Following the arguments similar to those given above, we have $N_v(t)\le \frac{\Lambda _v^{\sigma } }{\mu _v^{\sigma } }$ as $t\rightarrow \infty$. Therefore, system (4) has solutions in ${\mathcal {D}}$ and hence is positively invariant.

### The basic reproduction number of the model

The disease free equilibrium (DFE) of the model (4) is:

$$\begin{aligned} \begin{aligned} {\mathcal {D}}_{0}&=\left( S_{h}^{*}, V_h^{*}, E_{h1}^{*}, E_{h2}^{*}, E_{h12}^{*}, A_{h1}^{*}, A_{h2}^{*}, A_{h12}^{*}, I_{h1}^{*}, I_{h2}^{*}, I_{h12}^{*},Q_{h1}^{*},Q_{h2}^{*},Q_{h12}^{*},R_{h1}^{*},R_{h2}^{*},R_{h12}^{*},S_{v}^{*}, E_{v1}^{*},E_{v2}^{*},I_{v1}^{*},I_{v2}^{*}\right) \\&=\left( \frac{\Lambda _h^{\sigma }}{\mu _h^{\sigma }+\psi ^{\sigma }},\frac{\psi ^{\sigma }}{\mu _h^{\sigma }} \bigg (\frac{\Lambda _h^{\sigma }}{\mu _h^{\sigma }+\psi ^{\sigma }}\bigg ),0,0,0,0,0,0,0,0,0,0,0,0,0,0,0,\frac{\Lambda _v^{\sigma }}{\mu _v^{\sigma }},0,0,0,0\right) .\\ \end{aligned} \end{aligned}$$

Following the approach from^41^,

the “basic reproduction number” of the model (4), is given by

$\mathcal {{\mathcal {R}}}_{0} = \text {max}\{\mathcal {{\mathcal {R}}}_{01},\mathcal {{\mathcal {R}}}_{02}\}$, where $\mathcal {{\mathcal {R}}}_{01}$ and $\mathcal {{\mathcal {R}}}_{02}$ are the associated “reproduction numbers” for dengue strain-1 and dengue strain-2, respectively are given by

$$\begin{aligned} {\mathcal {R}}_{01} =\sqrt{\frac{\beta _{h1}^{\sigma }\beta _{v1}^{\sigma }A_{1}^{*}A_{2}^{*}\xi _{e1}^{\sigma } \omega _{v1}^{\sigma }(K_{3}+\xi _{a1}^{\sigma })}{K_{1}K_{2}K_{3}K_{5}K_{6}}}, \quad {\mathcal {R}}_{02} = \sqrt{\frac{\beta _{h2}^{\sigma }\beta _{v2}^{\sigma }A_{3}^{*}A_{2}^{*}\xi _{e2}^{\sigma } \omega _{v2}^{\sigma }(K_{9}+\xi _{a2}^{\sigma })}{K_{7}K_{8}K_{9}K_{11}K_{12}}}. \end{aligned}$$

Also, the following result can be established for the full model:

### Local asymptotic stability of the disease free equilibrium (DFE) of the model

#### Theorem 4.2

The system’s DFE, ${\mathcal {D}}_{0}$, is “locally asymptotically stable” (LAS) if ${\mathcal {R}}_{0}<1$, and unstable if ${\mathcal {R}}_{0}>1$.

## Existence, uniqueness and Ulam–Hyers stability of the complete model

### Existence

In this section, following the approach of^28^, we study the necessary conditions for existence of solution of the proposed model (4).

Consider a Banach space ${\mathbb {E}} = {\mathcal {C}}[{\mathcal {J}}, {\mathbb {R}}^{22}]$ equipped with the norm:

$$\Vert \varPhi \Vert = ~^{\sup }_{t \in {\mathcal {J}}} |\varPhi (t)|$$, where, $$|\varPhi (t)| = |\varPhi _1(t)|+|\varPhi _2(t)|+|\varPhi _3(t)|+\cdots +|\varPhi _{21}(t)|+|\varPhi _{22}(t)|$$.

The norm on ${\mathcal {C}}([{\mathcal {J}}, {\mathbb {R}}^{22}])$ or ${\mathcal {C}}([{\mathcal {J}}, {\mathbb {R}}])$ will be clear from its context. System (4) can be written in form of the Volterra integral equation given by

24 $$\begin{aligned} K(t)=K(0)+\frac{1}{\Gamma (\sigma )}\int _{0}^{t}(t-\wp )^{\sigma -1}{\mathcal {K}}(\wp ,K(\wp ))d\wp . \end{aligned}$$

Consider ${\textbf{B}} _{\eta }=\{\varPhi \in {\mathbb {E}}:\Vert \varPhi \Vert \le \eta \}$, where $\eta \ge |\varPhi _{0}|+\Omega \Vert \Psi \Vert$, $\varPhi _{0} \in {\mathbb {R}}^{22}$ and $\Omega = \frac{b^{\sigma }}{\Gamma (\omega +1)}$. Obviously ${\textbf{B}} _{\eta }$ is closed convex and bounded subset of E.

Define operators $P_{1},P_{2}: {\textbf {B}}_{\eta } \rightarrow {\mathbb {E}}$ by

$$\begin{aligned} \left( P_{1} \varPhi \right) (t){} & {} =\frac{1}{\Gamma (\sigma )} \int _{0}^{t}(t-\wp )^{\sigma -1} {\mathcal {K}}(\wp , \varPhi (\wp )) d \wp \quad \forall ~ t \in {\mathcal {J}}, \\ \left( P_{2} \varPhi \right) (t){} & {} =\varPhi _{0}, \quad \forall ~ t \in {\mathcal {J}}, \end{aligned}$$

respectively.

#### Lemma 5.1

The operator $\left( P_{2} \varPhi \right)$ is $\mu -$Lipschitz with constant $k \in (0,1).$

#### Proof

Since operator $\left( P_{2} \varPhi \right) (t)$ is constant so it is Lipschitz with Lipschitz constant $k \in (0,1).$ By lemma(1.4) $\left( P_{2} \varPhi \right) (t)$ is $\mu -$Lipschitz with constant $k \in (0,1).$

#### Lemma 5.2

If $|{\mathcal {K}}(t, \varPhi (t))| \le |\Psi (t)|, \quad for~ all \quad (t, \varPhi (t)) \in {\mathcal {J}} \times {\mathbb {R}}^{22}~~ and ~for~some ~~\Psi \in {\mathcal {C}}\left( {\mathcal {J}}, {\mathbb {R}}_{+}\right)$ with $\Vert \Psi \Vert =\sup _{t \in {\mathcal {J}}}|\Psi (t)|$. Then the operator $\left( P_{1} \varPhi \right)$ is $\mu -$Lipschitz with constant zero.

#### Proof

As the function ${\mathcal {K}}$ is continuous, so the operator $P_{1}$ is also continuous.

Now, for any $\varPhi \in {\textbf{B}}_{\eta }$, we have

$$\begin{aligned} \begin{aligned} \bigg \Vert \left( P_{1} \varPhi \right) \bigg \Vert&= ^{\sup }_{t\in {\mathcal {J}}} \bigg |P_{1}\varPhi (t)\bigg |\\ {}&= ^{\sup }_{t\in {\mathcal {J}}} \bigg | \frac{1}{\Gamma (\sigma )} \int _{0}^{t}(t-\wp )^{\sigma -1} {\mathcal {K}}(\wp , \varPhi (\wp )) d \wp \bigg |\\ {}&\le ^{\sup }_{t\in {\mathcal {J}}} \frac{1}{\Gamma (\sigma )} \int _{0}^{t}(t-\wp )^{\sigma -1} \bigg |\Psi (\wp )\bigg | d \wp \\&\le \frac{\bigg \Vert \Psi \bigg \Vert }{\Gamma (\sigma )} \sup _{t\in {\mathcal {J}}}\int _{0}^{t}(t-\wp ) ^{\sigma -1} d \wp \\&\le \frac{b^{\sigma }}{\Gamma (\sigma +1)}\bigg \Vert \Psi \bigg \Vert \\&= \Omega \bigg \Vert \Psi \bigg \Vert \le \eta . \end{aligned} \end{aligned}$$

Thus, $P_1 ({\textbf{B}}_{\eta }) \subset {\textbf{B}}_{\eta }$. As $\overline{P_1({\textbf{B}}_{\eta })}$ is bounded and closed. To apply the Arzela Ascoli theorem, we now prove that $\overline{P_1({\textbf{B}}_{\eta })}$ is equicontinuous.

For any $\varPhi \in {\textbf {B}}_{\eta }$, consider

$$\begin{aligned} \bigg |\left( P_{1} \varPhi \right) \left( t_{2}\right) -\left( P_{1} \varPhi \right) \left( t_{1}\right) \bigg | = \bigg | \frac{1}{\Gamma (\sigma )} \int _{0}^{t_2}(t_2-\wp )^{\sigma -1} {\mathcal {K}}(\wp , \varPhi (\wp )) d \wp - \frac{1}{\Gamma (\sigma )} \int _{0}^{t_1}(t_1-\wp )^{\sigma -1} {\mathcal {K}}(\wp , \varPhi (\wp )) d \wp \bigg | \\&\quad = \frac{1}{\Gamma (\sigma )} \bigg [\left| \int _{0}^{t_{1}}\left[ \left( t_{2}-\wp \right) ^{\sigma -1} - \left( t_{1}-\wp \right) ^{\sigma -1} \right] {\mathcal {K}}(\wp , \varPhi (\wp )) d \wp \right. \left. + \int _{t_{1}}^{t_{2}} \left( t_{2}-\wp \right) ^{\sigma -1} \mathcal { K}(\wp , \varPhi (\wp )) d \wp \right| \bigg ] \\&\quad \le \frac{\bigg \Vert \Psi \bigg \Vert }{\Gamma (\sigma +1)}\bigg [(t_{2}^{\sigma }-t_{1}^{ \sigma })\bigg ]. \end{aligned}$$

Clearly, the right hand side of the above inequality vanishes as $t_{2} \rightarrow t_{1}$. Thus, $P_{1}{} {\textbf {B}}_{\eta }$ is equicontinuous and so it $\overline{P_1 ({\textbf {B}}_{\eta })}$. Hence, $\overline{P_1 ({\textbf {B}}_{\eta })}$ being closed, bounded and equicontinuous is compact which gives that $P_1$ is a compact operator. Thus by lemma (1.3) $P_1$ is $\mu -$Lipschitz with constant 0.

#### Theorem 5.1

Assume that the conditions of the lemmas (5.1) and (5.2) hold. Then the integral equation has at least one solution in *E* Moreover, the set of solutions of (24) is bounded in *E*.

#### Proof

: By Lemma 5.1, $P_2$ is $\mu$-Lipschitz with constant *k*, and by Lemma 5.2, $P_1$ is $\mu$-Lipschitz with constant 0 . Hence ${\textbf{P}}=\mathbf {P_1}+\mathbf {P_2}$ is $\mu$-Lipschitz with constant *k* and hence $\mathbf {P_1}+\mathbf {P_2}$ is $\mu$-condensing. Define

$$\begin{aligned} G=\{\phi \in E: h \in [0,1] ~\text {such that}~ \phi =h {\textbf{P}}(\phi )\} \end{aligned}$$

Let $\phi \in E$, then we have

$$\begin{aligned} \Vert \phi \Vert =\Vert h{\textbf{P}}(\phi )\Vert =h\Vert {\textbf{P}}(\phi )\Vert =h\Vert K(0)+\frac{1}{\Gamma (\sigma )} \int _{0}^{t}(t-s)^{\sigma -1}\kappa (t,\phi (t))ds\Vert \le h[\phi _{0}+\frac{b^\sigma \Vert \Psi \Vert }{\Gamma (\sigma +1)}]\Vert \le \eta . \end{aligned}$$

Thus $G \subseteq B_{\eta }$ and hence bounded and contained in $B_{r}(0)$. By Theorem (1.1), an operator ${\textbf{P}}$ has atleast one solution.

### Uniqueness

#### Theorem 5.2

Suppose that the function ${\mathcal {K}} \in {\mathcal {C}}([{\mathcal {J}}, {\mathbb {R}}^{22}])$ satisfy the Lipschitz condition

25 $$\begin{aligned} \left| {\mathcal {K}}\left( t, \varPhi _{1}(t)\right) -{\mathcal {K}}\left( t, \varPhi _{2}(t)\right) \right| \le {\mathcal {L}}_{{\mathcal {K}}}\left| \varPhi _{1}(t)-\varPhi _{2}(t)\right| , \end{aligned}$$

for all $t \in$ ${\mathcal {J}}$ and each $\varPhi _{1}, \varPhi _{2} \in {\mathbb {E}}$, $~ {\mathcal {L}}_{{\mathcal {K}}}>0$. Then system (4), or its equivalent form (24) has unique solution whenever $\Omega {\mathcal {L}}_{{\mathcal {K}}}<1$.

#### Proof

Consider the operator $P: {\mathbb {E}} \rightarrow {\mathbb {E}}$ defined by

$$\begin{aligned} (P \varPhi )(t)=\varPhi _{0} +\frac{1}{\Gamma (\sigma )} \int _{0}^{t} {\mathcal {K}}(\wp ,\varPhi (t))(t-\wp )^{\sigma -1} d \wp . \end{aligned}$$

Now for any $\varPhi _{1}, \varPhi _{2} \in {\mathbb {E}}$, we get

$$\begin{aligned} \begin{aligned} \bigg \Vert \left( P \varPhi _{1}\right) -\left( P \varPhi _{2}\right) \bigg \Vert&\le \sup _{t \in {\mathcal {J}}}\bigg [\bigg |\varPhi _{0}+ \frac{1}{\Gamma (\sigma )} \int _{0}^{t}(t-\wp )^{\sigma -1} {\mathcal {K}}\left( \wp , \varPhi _{1}(\wp )\right) d \wp \\&\quad -\bigg ( \varPhi _{0}+ \frac{1}{\Gamma (\sigma )} \int _{0}^{t}(t-\wp )^{\sigma -1} {\mathcal {K}}\left( \wp , \varPhi _{2}(\wp )d \wp \right) \bigg )\bigg |\bigg ] \\&\le \sup _{t \in {\mathcal {J}}}\frac{1}{\Gamma (\sigma )} \int _{0}^{t}(t-\wp )^{\sigma -1} \bigg | {\mathcal {K}}\left( \wp , \varPhi _{1}(\wp )\right) \\&\quad -{\mathcal {K}}\left( \wp , \varPhi _{2}(\wp )\right) \bigg | d \wp \\&\le \sup _{t \in {\mathcal {J}}}\frac{{\mathcal {L}}_{{\mathcal {K}}}}{\Gamma (\sigma )} \int _{0}^{t}(t-\wp )^{\sigma -1}\bigg |\varPhi _{1}(\wp )-\varPhi _{2}(\wp )\bigg | d \wp \\&\le \frac{{\mathcal {L}}_{{\mathcal {K}}} \bigg \Vert \varPhi _{1}-\varPhi _{2}\bigg \Vert }{\Gamma (\sigma )}\sup _{t \in {\mathcal {J}}} \int _{0}^{t}(t-\wp )^{\sigma -1} d \wp \\&\le \frac{b^{\sigma }}{\Gamma (\sigma +1)}{\mathcal {L}}_{{\mathcal {K}}}\bigg \Vert \varPhi _{1}-\varPhi _{2}\bigg \Vert \\&= \Omega {\mathcal {L}}_{{\mathcal {K}}}\bigg \Vert \varPhi _{1}(t)-\varPhi _{2}(t)\bigg \Vert . \end{aligned} \end{aligned}$$

This implies that *P* is a contraction.

As $P(\varPhi )(t)=P_{1}(\varPhi )(t)+P_{2}(\varPhi )(t)$, so $P {\textbf{B}}_{\eta } \subset {\textbf{B}}_{\eta }$. Since the set $\textbf{ B}_{\eta }$ is closed, it follows from Banach contraction principle that the proposed model possess a unique solution.

## Ulam–Hyers stability

The stability result for the fractional system is now studied in the frame-work of Ulam-Hyers (UH) stability^42,43^.Let ${\mathbb {E}} = {\mathcal {C}}({\mathcal {J}},{\mathbb {R}}^{22})$ be space of “continuous functions” from ${\mathcal {J}}$ to ${\mathbb {R}}^{22}$ coupled with the norm $\Vert \varPhi \Vert = ^{sup}_{t \in {\mathcal {J}}} \left| \varPhi (t) \right|$, where ${\mathcal {J}}=[0,b]$.

### Definition 6.1

The model (4) or its transformed version given by

26 $$\begin{aligned} {\left\{ \begin{array}{ll} ^{C}D^{\sigma }_{t}{\varPhi }(t) &{} ={\mathcal {K}}(t,{\varPhi }(t)), \\ {\varPhi }(0) &{} =\varPhi _0, \end{array}\right. } \end{aligned}$$

is UH stable if $\exists$ $k>0$ such that for any $\varepsilon >0$ and the given solution of (26) satisfying the following inequality

27 $$\begin{aligned} \Vert ^{C}D^\sigma {\bar{\varPhi }}(t) - {\mathcal {K}} (t,{\bar{\varPhi }}(t) \Vert \le \varepsilon , ~ ~ t \in {\mathcal {J}}, ~ \varepsilon = \max (\varepsilon _i)^T, ~ i = 1, 2, \dots 22. \end{aligned}$$

$\exists$ unique solution $\varPhi \in {\mathbb {E}}$ of system (26) in such a way that

$$\begin{aligned} \Vert {\bar{\varPhi }}(t) - \varPhi (t) \Vert \le k \varepsilon , ~ t \in {\mathcal {J}}, ~ ~ k = \max (k_j)^T, ~ j = 1, 2, \dots 22. \end{aligned}$$

### Definition 6.2

System (26) is “generalized UH stable” if $\exists$ a continuous function $\phi : {\mathbb {R}}^+ \rightarrow {\mathbb {R}}^+$ with $\phi (0) = 0$ such that for any other solution ${\bar{\varPhi }} \in \mathbb {E }$ of the inequality (27), there exists a unique solution $\varPhi \in {\mathbb {E}}$ satisfying the following:

$$\begin{aligned} \Vert {\bar{\varPhi }}(t) - \varPhi (t) \Vert \le \phi (\varepsilon ), ~ t \in {\mathcal {J}}, ~ \phi = \max (\phi _j)^T, ~ j = 1, 2, \dots 22. \end{aligned}$$

### Remark 6.1

A function ${\bar{\varPhi }} \in {\mathbb {E}}$ satisfies the inequality (27) if and only if there exists a function $h \in {\mathbb {E}}$ having the following properties:

i. $$\left\| h(t) \right\| \le \varepsilon , ~ t \in {\mathcal {J}}$$.
ii. $$^{C}D^\sigma {\bar{\varPhi }}(t) = {\mathcal {K}}(t,{\bar{\varPhi }}(t) + h(t)$$ ,   $$t\in {\mathcal {J}}$$.

### Lemma 6.1

If ${\bar{\varPhi }} \in {\mathbb {E}}$ holds for system (27), then ${\bar{\varPhi }}$ also holds for the following:

28 $$\begin{aligned} \left| {\bar{\varPhi }}(t) - \left( {\bar{\varPhi }}_0 + \frac{1}{\Gamma (\sigma )} \int ^t_0\left( t-\wp \right) ^{\sigma -1}{\mathcal {K}}(\wp , {\bar{\varPhi }}(\wp ))d\wp \right) \right| \le \Omega \varepsilon \end{aligned}$$

### Proof

Using (ii.) of the Remark 6.1, we have $^{C} D^\sigma {\bar{\varPhi }} (t) = {\mathcal {K}}(t,{\bar{\varPhi }}(t) ) + h(t)$,   $t\in {\mathcal {J}}$,which on applying Caputo integral gives that

29 $$\begin{aligned} {\bar{\varPhi }}(t) = {\bar{\varPhi }}_0 + \frac{1}{\Gamma (\sigma )} \int ^t_0\left( t-\wp \right) ^{\sigma -1}{\mathcal {K}}(\wp , {\bar{\varPhi }}(\wp ) )d\wp +\frac{1}{\Gamma (\sigma )}\int ^t_0\left( t-\wp \right) ^{\sigma -1}h(\wp )d\wp \end{aligned}$$

Re-arranging and taking the norm on the both sides and applying the item (i.) of Remark 6.1, we obtain that

$$\begin{aligned} \left| {\bar{\varPhi }}(t) - \left( {\bar{\varPhi }}_0 + \frac{1}{\Gamma (\sigma )}\int ^t_0\left( t-\wp \right) ^{\sigma -1}{\mathcal {K}} (\wp , {\bar{\varPhi }}(\wp ) )d\wp \right) \right| \\&\quad \le \frac{1}{\Gamma (\sigma )}\int ^t_0\left( t-\wp \right) ^{\sigma -1}\left| h(\wp )\right| d\wp \\&\quad \le \left( \frac{b^{\sigma }}{\Gamma (\sigma +1)}\right) \varepsilon \le \Omega \varepsilon . \\ \end{aligned}$$

### Theorem 6.1

For all $\varPhi \in {\mathbb {E}}$ and the Lipschitz mapping ${\mathcal {K}}:{\mathcal {J}} \times \mathbb {R }^{22} \rightarrow {\mathbb {R}}^{22}$ with Lipschitz constant ${\mathcal {L}}_{{\mathcal {K}}}>0$ and $1 - \Omega {\mathcal {L}}_{{\mathcal {K}}}>0$, where $\Omega = \frac{b^\sigma }{\Gamma (\sigma +1)}$, the model (26) is generalized UH stable.

### Proof

If ${\bar{\varPhi }} \in {\mathbb {E}}$ satisfies the inequality given by (27) and $\varPhi \in {\mathbb {E}}$ is a unique solution of (26). Then $\forall ~ \varepsilon > 0, ~ t \in {\mathcal {J}}$ together with Lemma 6.1, we have

$$\begin{aligned} \begin{aligned} \left\| {\bar{\varPhi }}(t) - \varPhi (t)\right\|&= ~ ^{\sup }_{t\in {\mathcal {J}}} \left| {\bar{\varPhi }}_0 + \frac{1}{\Gamma (\sigma )} \int ^t_0\left( t-\wp \right) ^{\sigma -1}{\mathcal {K}}(\wp , {\bar{\varPhi }}(\wp ) )d\wp + \frac{1}{\Gamma (\sigma )}\int ^t_0\left( t-\wp \right) ^{\sigma -1}h(\wp )d\wp \right. \\&\left. \quad - \left( \varPhi _0 + \frac{1}{\Gamma (\sigma )}\int ^t_0\left( t-\wp \right) ^{\sigma -1}{\mathcal {K}} (\wp , \varPhi (\wp ))d\wp \right) \right| \\&\le ~ ^{\sup }_{t\in {\mathcal {J}}} \left| {\bar{\varPhi }}_{0} - \varPhi _0 \right| + ~ ^{\sup }_{t\in {\mathcal {J}}} \bigg [|h(t)| \left( \frac{1}{\Gamma (\sigma )}\int ^t_0\left( t-\wp \right) ^{\sigma -1}d\wp \right) \bigg ] \\&\quad + ~ ^{\sup }_{t\in {\mathcal {J}}} \frac{1}{\Gamma (\sigma )} \int ^t_0\left( t-\wp \right) ^{\sigma -1}\left| {\mathcal {K}}(t, {\bar{\varPhi }} (t)) - {\mathcal {K}}(t, \varPhi (t))\right| d\wp \\&\le \Omega \varepsilon + \frac{{\mathcal {L}}_{{\mathcal {K}}}\left\| \bar{ \varPhi } - \varPhi \right\| }{\Gamma (\sigma )}~^{\sup }_{t\in {\mathcal {J}}}\int ^t_0\left( t-\wp \right) ^{\sigma -1}d\wp \\&\le \Omega \varepsilon + \left( \frac{b^{\sigma }}{\Gamma (\sigma +1)} \right) {\mathcal {L}}_{{\mathcal {K}}} \left\| {\bar{\varPhi }} - \varPhi \right\| \\&= \Omega \varepsilon + \Omega {\mathcal {L}}_{{\mathcal {K}}} \left\| {\bar{\varPhi }}(t) - \varPhi (t) \right\| . \end{aligned} \end{aligned}$$

Thus, we have

30 $$\begin{aligned} \Vert {\bar{\varPhi }} - \varPhi \Vert \le k \varepsilon , \end{aligned}$$

where, $$k = \frac{\Omega }{1 - \Omega {\mathcal {L}}_{{\mathcal {K}}}}$$.

Thus, if we take $\phi (\varepsilon ) = k \varepsilon$, then $\phi (0) = 0$ and hence the system (26) is both Ulam Hyers (UH) and generalized UH stable.

## Nonstandard finite difference scheme

In order to analyze the disease’s spread, we applied a nonstandard finite difference (NSFD) scheme^44^ for the model that can ensure the solution’s positivity and displays the right asymptotic behavior. Consider the Caputo derivative

$$\begin{aligned} ^{c}D_{0+}^{\sigma } f(t)=\frac{1}{\Gamma (1-\sigma )}\int _{0}^{t}f^{'}(\theta )(t-\theta )^{-\sigma }d\theta . \end{aligned}$$

The discretization of the domain [0, *T*] is given by $t_{j} = jp,~ j=0,1,2,\cdot \cdot \cdot , N$, where *p* is the step size $p=\frac{T}{N}$ and T is the final time. The equation becomes for $t=t_{j+1}$

31 $$\begin{aligned} ^{c}D_{0+}^{\sigma } f(t)|_{t=t_{j+1}}=\frac{1}{\Gamma (1-\sigma )}\sum _{k=0}^{j}\int _{t_{k}}^{t_{k+1}}f^{'} (\theta )(t_{j+1}-\theta )^{-\sigma }d\theta . \end{aligned}$$

The approximation of $f^{'}(\theta )$ is given as

$$\begin{aligned} \frac{d f(\theta )}{d \theta }=f^{'}(\theta )=\frac{f^{k+1}-f^{k}}{\Psi (p)}, \end{aligned}$$

where denominator function $\Psi (p)$ is defined as

$$\begin{aligned} \Psi (p)=\frac{p^{1-\sigma }(1-E_{\sigma }(-(\mu _h^{\sigma } p)^{\sigma }))}{(E_{\sigma }(-(\mu _h^{\sigma } p)^{\sigma }))\Gamma (2-\sigma )\mu _h^{\sigma }}, \end{aligned}$$

so that equation (31) becomes

32 $$\begin{aligned} ^{c}D_{0+}^{\sigma }f(t)|_{t=t_{j+1}}=\frac{1}{\Gamma (1-\sigma )}\sum _{k=0}^{j}\frac{f^{k+1}-f^{k}}{\Psi (p)}\int _{t_{k}}^{t_{k+1}}(t_{j+1}-\theta )^{-\sigma }d\theta , \end{aligned}$$

which further gives

33 $$\begin{aligned} ^{c}D_{0+}^{\sigma }f(t)|_{t=t_{j+1}}=\frac{1}{\Gamma (2-\sigma )}\sum _{k=0}^{j}\frac{f^{k+1}-f^{k}}{\Psi (p)}A_{\sigma ,j}^{k}, \end{aligned}$$

where $$A_{\sigma ,j}^{k}=p^{1-\sigma }\bigg ((j-k+1)^{1-\sigma }-(j-k)^{1-\sigma }\bigg ).$$

Now following the^44^ and using (33), the NSFD scheme for the model 4 is given by the following equations:

34 $$\begin{aligned}{} & {} \begin{aligned} S_{h}^{j+1}&=\frac{p^{1-\sigma }S_{h}^{j}+\Gamma (2-\sigma )\Psi (p)(\Lambda _h^{\sigma }+\delta _{h1}^{\sigma } R_{h1}^{j} + \delta _{h2}^{\sigma }R_{h2}^{j}+\delta _{h12}^{\sigma }R_{h12}^{j})-\sum _{k=0}^{j-1} (S_{h}^{k+1}-S_{h}^{k})A_{\sigma ,j}^{k}}{p^{1-\sigma }+\Psi (p)\Gamma (2-\sigma )(\frac{\beta _{h1}^{\sigma } I_{v1}^{j}}{1+\alpha _1^{\sigma } I_{v1}^{j}} + \frac{\beta _{h2}^{\sigma } I_{v2}^{j}}{1+\alpha _2^{\sigma } I_{v2}^{j}}+ (\mu _{h}^{\sigma } + \psi ^{\sigma }))},\\ V_{h}^{j+1}&=\frac{p^{1-\sigma }V_{h}^{j}+\Gamma (2-\sigma )\Psi (p)(\psi ^{\sigma } S_{h}^{j+1})-\sum _{k=0}^{j-1}(V_{h}^{k+1}-V_{h}^{k})A_{\sigma ,j}^{k}}{p^{1-\sigma } +\Psi (p)\Gamma (2-\sigma )((1-\phi _1^{\sigma }) \frac{\beta _{h1}^{\sigma } I_{v1}^{j}}{1+\alpha _1^{\sigma } I_{v1}^{j}} + (1-\phi _2^{\sigma }) \frac{\beta _{h2}^{\sigma } I_{v2}^{j}}{1+\alpha _2^{\sigma } I_{v2}^{j}} + \mu _{h}^{\sigma })},\\ E_{h1}^{j+1}&=\frac{p^{1-\sigma }E_{h1}^{j}+\Gamma (2-\sigma )\Psi (p)(\frac{\beta _{h1}^{\sigma } I_{v1}^{j}}{1+\alpha _1^{\sigma } I_{v1}^{j}} [S_{h}^{j+1} + (1-\phi _1^{\sigma })V_{h}^{j+1}])-\sum _{k=0}^{j-1}(E_{h1}^{k+1}-E_{h1}^{k})A_{\sigma ,j}^{k}}{p^{1-\sigma }+\Psi (p)\Gamma (2-\sigma )((\mu _{h}^{\sigma } + \xi _{e1}^{\sigma }) + \frac{\beta _{h2}^{\sigma } I_{v2}^{j}}{1+\alpha _2^{\sigma } I_{v2}^{j}})},\\ E_{h2}^{j+1}&=\frac{p^{1-\sigma }E_{h2}^{j}+\Gamma (2-\sigma )\Psi (p)(\frac{\beta _{h2}^{\sigma } I_{v2}^{j}}{1+\alpha _2^{\sigma } I_{v2}^{j}} [S_{h}^{j+1} + (1-\phi _2^{\sigma })V_{h}^{j+1}])-\sum _{k=0}^{j-1}(E_{h2}^{k+1}-E_{h2}^{k})A_{\sigma ,j}^{k}}{p^{1-\sigma }+\Psi (p)\Gamma (2-\sigma )((\mu _{h}^{\sigma } + \xi _{e2}^{\sigma }) + \frac{\beta _{h1}^{\sigma } I_{v1}^{j}}{1+\alpha _1^{\sigma } I_{v1}^{j}})},\\ E_{h12}^{j+1}&=\frac{p^{1-\sigma }E_{h12}^{j}+\Gamma (2-\sigma )\Psi (p)(\frac{\beta _{h2}^{\sigma } I_{v2}^{j}}{1+\alpha _2^{\sigma } I_{v2}^{j}} E_{h1}^{j+1} + \frac{\beta _{h1}^{\sigma } I_{v1}^{j}}{1+\alpha _1^{\sigma } I_{v1}^{j}} E_{h2}^{j+1})-\sum _{k=0}^{j-1}(E_{h12}^{k+1}-E_{h12}^{k})A_{\sigma ,j}^{k}}{p^{1-\sigma } +\Psi (p)\Gamma (2-\sigma )((\mu _{h}^{\sigma } + \xi _{e12}^{\sigma }))},\\ A_{h1}^{j+1}&=\frac{p^{1-\sigma }A_{h1}^{j}+\Gamma (2-\sigma )\Psi (p)(\xi _{e1}^{\sigma } E_{h1}^{j+1})-\sum _{k=0}^{j-1}(A_{h1}^{k+1}-A_{h1}^{k})A_{\sigma ,j}^{k}}{p^{1-\sigma } +\Psi (p)\Gamma (2-\sigma )((\mu _{h}^{\sigma } + \xi _{a1}^{\sigma } + \zeta _{a1}^{\sigma } + \theta _{a1}^{\sigma })+ \frac{\beta _{h2}^{\sigma } I_{v2}^{j}}{1+\alpha _2^{\sigma } I_{v2}^{j}})},\\ A_{h2}^{j+1}&=\frac{p^{1-\sigma }A_{h2}^{j}+\Gamma (2-\sigma )\Psi (p)(\xi _{e2}^{\sigma } E_{h2}^{j+1})-\sum _{k=0}^{j-1}(A_{h2}^{k+1}-A_{h2}^{k})A_{\sigma ,j}^{k}}{p^{1-\sigma } +\Psi (p)\Gamma (2-\sigma )((\mu _{h}^{\sigma } + \xi _{a2}^{\sigma } + \zeta _{a2}^{\sigma } + \theta _{a2}^{\sigma })+ \frac{\beta _{h1}^{\sigma } I_{v1}^{j}}{1+\alpha _1^{\sigma } I_{v1}^{j}})},\\ A_{h12}^{j+1}&=\frac{p^{1-\sigma }A_{h12}^{j}+\Gamma (2-\sigma )\Psi (p)(\xi _{e12}^{\sigma } E_{h12}^{j+1}+\frac{\beta _{h2}^{\sigma } I_{v2}^{j}}{1+\alpha _2^{\sigma } I_{v2}^{j}} A_{h1}^{j+1} + \frac{\beta _{h1}^{\sigma } I_{v1}^{j}}{1+\alpha _1^{\sigma } I_{v1}^{j}} A_{h2}^{j+1})-\sum _{k=0}^{j-1}(A_{h12}^{k+1}-A_{h12}^{k})A_{\sigma ,j}^{k}}{p^{1-\sigma } +\Psi (p)\Gamma (2-\sigma )(\mu _{h}^{\sigma } + \xi _{a12}^{\sigma } + \zeta _{a12}^{\sigma } + \theta _{a12}^{\sigma })},\\ I_{h1}^{j+1}&=\frac{p^{1-\sigma }I_{h1}^{j}+\Gamma (2-\sigma )\Psi (p)(\xi _{a1}^{\sigma } A_{h1}^{j+1})-\sum _{k=0}^{j-1}(I_{h1}^{k+1}-I_{h1}^{k})A_{\sigma ,j}^{k}}{p^{1-\sigma }+\Psi (p) \Gamma (2-\sigma )((\mu _{h}^{\sigma } + \zeta _{i1}^{\sigma } + \theta _{i1}^{\sigma } + \eta _{i1}^{\sigma }) + \frac{\beta _{h2}^{\sigma } I_{v2}^{j}}{1+\alpha _2^{\sigma } I_{v2}^{j}})},\\ I_{h2}^{j+1}&=\frac{p^{1-\sigma }I_{h2}^{j}+\Gamma (2-\sigma )\Psi (p)(\xi _{a2}^{\sigma } A_{h2}^{j+1})-\sum _{k=0}^{j-1}(I_{h2}^{k+1}-I_{h2}^{k})A_{\sigma ,j}^{k}}{p^{1-\sigma }+\Psi (p) \Gamma (2-\sigma )((\mu _{h}^{\sigma } + \zeta _{i2}^{\sigma } + \theta _{i2}^{\sigma } + \eta _{i2}^{\sigma })+\frac{\beta _{h1}^{\sigma } I_{v1}^{j}}{1+\alpha _1^{\sigma } I_{v1}^{j}})},\\ I_{h12}^{j+1}&=\frac{p^{1-\sigma }I_{h12}^{j}+\Gamma (2-\sigma )\Psi (p)(\xi _{a12}^{\sigma } A_{h12}^{j+1}+\frac{\beta _{h2}^{\sigma } I_{v2}^{j}}{1+\alpha _2^{\sigma } I_{v2}^{j}} I_{h1}^{j+1} + \frac{\beta _{h1}^{\sigma } I_{v1}^{j}}{1+\alpha _1^{\sigma } I_{v1}^{j}} I_{h2}^{j+1})-\sum _{k=0}^{j-1}(I_{h12}^{k+1}-I_{h12}^{k})A_{\sigma ,j}^{k}}{p^{1-\sigma }+\Psi (p) \Gamma (2-\sigma )((\mu _{h}^{\sigma } + \zeta _{i12}^{\sigma } + \theta _{i12}^{\sigma } + \eta _{i12}^{\sigma }))},\\ Q_{h1}^{j+1}&=\frac{p^{1-\sigma }Q_{h1}^{j}+\Gamma (2-\sigma )\Psi (p)(\eta _{i1}^{\sigma } I_{h1}^{j+1})-\sum _{k=0}^{j-1}(Q_{h1}^{k+1}-Q_{h1}^{k})A_{\sigma ,j}^{k}}{p^{1-\sigma }+\Psi (p) \Gamma (2-\sigma )((\mu _{h}^{\sigma } + \zeta _{h1}^{\sigma } + \theta _{h1}^{\sigma }))}.\\ \end{aligned} \end{aligned}$$

35 $$\begin{aligned}{} & {} \begin{aligned} Q_{h2}^{j+1}&=\frac{p^{1-\sigma }Q_{h2}^{j}+\Gamma (2-\sigma )\Psi (p)(\eta _{i2}^{\sigma } I_{h2}^{j+1})-\sum _{k=0}^{j-1}(Q_{h2}^{k+1}-Q_{h2}^{k})A_{\sigma ,j}^{k}}{p^{1-\sigma } +\Psi (p)\Gamma (2-\sigma )((\mu _{h}^{\sigma } + \zeta _{h2}^{\sigma } + \theta _{h2}^{\sigma }))},\\ Q_{h12}^{j+1}&=\frac{p^{1-\sigma }Q_{h12}^{j}+\Gamma (2-\sigma )\Psi (p)(\eta _{i12}^{\sigma } I_{h12}^{j+1})-\sum _{k=0}^{j-1}(Q_{h12}^{k+1}-Q_{h12}^{k})A_{\sigma ,j}^{k}}{p^{1-\sigma } +\Psi (p)\Gamma (2-\sigma )(\mu _{h}^{\sigma } + \zeta _{h12}^{\sigma } + \theta _{h12}^{\sigma })},\\ R_{h1}^{j+1}&=\frac{p^{1-\sigma }R_{h1}^{j}+\Gamma (2-\sigma )\Psi (p)(\zeta _{a1}^{\sigma } A_{h1}^{j+1} + \zeta _{i1}^{\sigma } I_{h1}^{j+1} + \zeta _{h1}^{\sigma } Q_{h1}^{j+1})-\sum _{k=0}^{j-1}(R_{h1}^{k+1}-R_{h1}^{k})A_{\sigma ,j}^{k}}{p^{1-\sigma } +\Psi (p)\Gamma (2-\sigma )(\mu _{h}^{\sigma } + \delta _{h1}^{\sigma })},\\ R_{h2}^{j+1}&=\frac{p^{1-\sigma }R_{h2}^{j}+\Gamma (2-\sigma )\Psi (p)(\zeta _{a2}^{\sigma } A_{h2}^{j+1} + \zeta _{i2}^{\sigma } I_{h2}^{j+1} + \zeta _{h2}^{\sigma } Q_{h2}^{j+1})-\sum _{k=0}^{j-1}(R_{h2}^{k+1}-R_{h2}^{k})A_{\sigma ,j}^{k}}{p^{1-\sigma } +\Psi (p)\Gamma (2-\sigma )(\mu _{h}^{\sigma } + \delta _{h2}^{\sigma })},\\ R_{h12}^{j+1}&=\frac{p^{1-\sigma }R_{h12}^{j}+\Gamma (2-\sigma )\Psi (p)(\zeta _{a12}^{\sigma } A_{h12}^{j+1} + \zeta _{i12}^{\sigma } I_{h12}^{j+1} + \zeta _{h12}^{\sigma } Q_{h12}^{j+1})-\sum _{k=0}^{j-1}(R_{h12}^{k+1}-R_{h12}^{k})A_{\sigma ,j}^{k}}{p^{1-\sigma } +\Psi (p)\Gamma (2-\sigma )(\mu _{h}^{\sigma } + \delta _{h12}^{\sigma })},\\ S_{v}^{j+1}&=\frac{p^{1-\sigma }S_{v}^{j}+\Gamma (2-\sigma )\Psi (p)(\Lambda _v^{\sigma }) -\sum _{k=0}^{j-1}(S_{v}^{k+1}-S_{v}^{k})A_{\sigma ,j}^{k}}{p^{1-\sigma }+\Psi (p)\Gamma (2-\sigma ) (\frac{\beta _{v1}^{\sigma }(A_{h1}^{j+1} + I_{h1}^{j+1} + A_{h12}^{j+1} + I_{h12}^{j+1})}{1+\gamma _1^{\sigma } A_{h1}^{j+1} + \gamma _2^{\sigma } I_{h1}^{j+1} + \gamma _3^{\sigma } A_{h12}^{j+1} + \gamma _4^{\sigma } I_{h12}^{j+1}} + \frac{\beta _{v2}^{\sigma }(A_{h2}^{j+1} + I_{h2}^{j+1} + A_{h12}^{j+1} + I_{h12}^{j+1})}{1+\gamma _1^{\sigma } A_{h2}^{j+1} + \gamma _2^{\sigma } I_{h2}^{j+1} + \gamma _3^{\sigma } A_{h12}^{j+1} + \gamma _4^{\sigma } I_{h12}^{j+1}}+ \mu _{v}^{\sigma })},\\ E_{v1}^{j+1}&=\frac{p^{1-\sigma }E_{v1}^{j}+\Gamma (2-\sigma )\Psi (p)(\frac{\beta _{v1}^{\sigma }(A_{h1}^{j+1} + I_{h1}^{j+1} + A_{h12}^{j+1} + I_{h12}^{j+1})}{1+\gamma _1^{\sigma } A_{h1}^{j+1} + \gamma _2^{\sigma } I_{h1}^{j+1} + \gamma _3^{\sigma } A_{h12}^{j+1} + \gamma _4^{\sigma } I_{h12}^{j+1}} S_{v}^{j+1})-\sum _{k=0}^{j-1}(E_{v1}^{k+1}-E_{v1}^{k})A_{\sigma ,j}^{k}}{p^{1-\sigma } +\Psi (p)\Gamma (2-\sigma )(\mu _{v}^{\sigma } + \omega _{v1}^{\sigma })},\\ E_{v2}^{j+1}&=\frac{p^{1-\sigma }E_{v2}^{j}+\Gamma (2-\sigma )\Psi (p)(\frac{\beta _{v2}^{\sigma }(A_{h2}^{j+1} + I_{h2}^{j+1} + A_{h12}^{j+1} + I_{h12}^{j+1})}{1+\gamma _1^{\sigma } A_{h2}^{j+1} + \gamma _2^{\sigma } I_{h2}^{j+1} + \gamma _3^{\sigma } A_{h12}^{j+1} + \gamma _4^{\sigma } I_{h12}^{j+1}} S_{v}^{j+1})-\sum _{k=0}^{j-1}(E_{v2}^{k+1}-E_{v2}^{k})A_{\sigma ,j}^{k}}{p^{1-\sigma }+\Psi (p) \Gamma (2-\sigma )(\mu _{v}^{\sigma } + \omega _{v2}^{\sigma })},\\ I_{v1}^{j+1}&=\frac{p^{1-\sigma }I_{v1}^{j}+\Gamma (2-\sigma )\Psi (p)(\omega _{v1}^{\sigma }E_{v1} ^{j+1})-\sum _{k=0}^{j-1}(I_{v1}^{k+1}-I_{v1}^{k})A_{\sigma ,j}^{k}}{p^{1-\sigma }+\Psi (p) \Gamma (2-\sigma )(\mu _{v}^{\sigma } + \rho _{v1}^{\sigma })},\\ I_{v2}^{j+1}&=\frac{p^{1-\sigma }I_{v2}^{j}+\Gamma (2-\sigma )\Psi (p)(\omega _{v2}^{\sigma } E_{v2}^{j+1})-\sum _{k=0}^{j-1}(I_{v2}^{k+1}-I_{v2}^{k})A_{\sigma ,j}^{k}}{p^{1-\sigma } +\Psi (p)\Gamma (2-\sigma )(\mu _{v}^{\sigma } + \rho _{v2}^{\sigma })}.\\ \end{aligned} \end{aligned}$$

## Model fitting and numerical assessment

**Figure 2:**
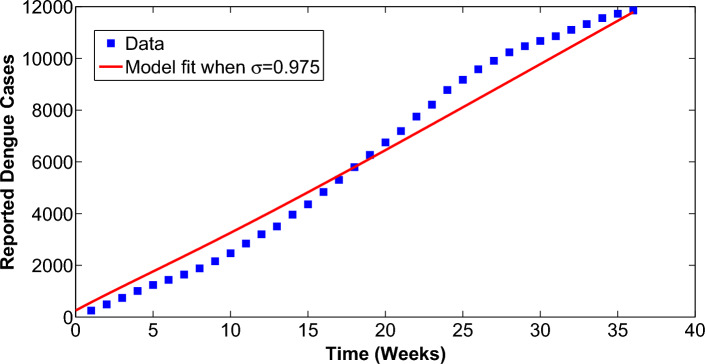
Fitting the model to data.

Demographic data related to Brazil have been used for the simulations. The initial conditions are set as: $S_h(0) = 3{,}600{,}000$, $V_{h}(0) = 400{,}000$, $E_{h1}(0)=125$, $E_{h2}(0)=1,26$, $E_{h12}(0)=0$, $A_{h1}(0)=500$, $A_{h2}(0)=500$, $A_{h12}(0)=0$, $I_{h1}(0)=500$, $I_{h2}(0)=500$, $I_{h12}(0)=0$, $Q_{h1}(0)=500$, $Q_{h2}(0)=600$,$Q_{h12}(0)=0$, $R_{h1}(0)=500$, $R_{h2}(0)=500$, $R_{h2}(0)=0$, $S_{v}(0)=48{,}000$, $E_{v1}(0)=600$, $E_{v2}(0)=600$, $I_{v1}(0)=1000$, $I_{v2}(0)=1000$. For the fitting of model to data available records for reported Dengue cases in Espirito Santo Brazil for 36 weeks^45^, the fractional model is fitted to real data. The fitting, which is shown in Fig. 2 reveals that our model behaves very well to data when the order of the Caputo derivative is taken as: $\sigma =0.975$.

### Sensitivity analysis of reproduction numbers

**Figure 3:**
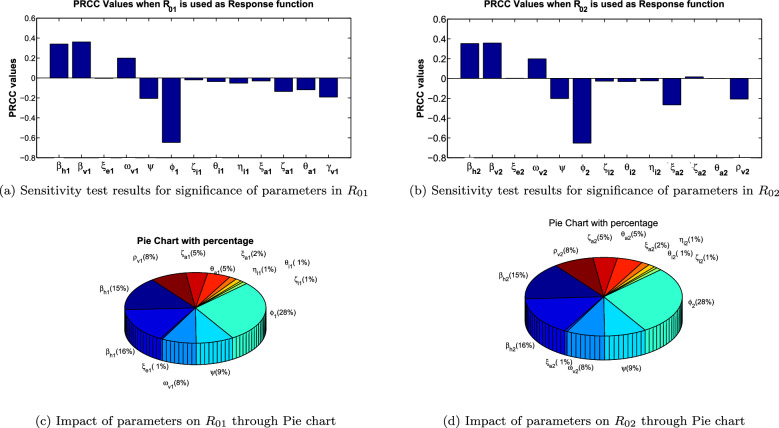
Illustration of the influence of parameters on reproduction number through PRCC and Pie Chart.

**Figure 4:**
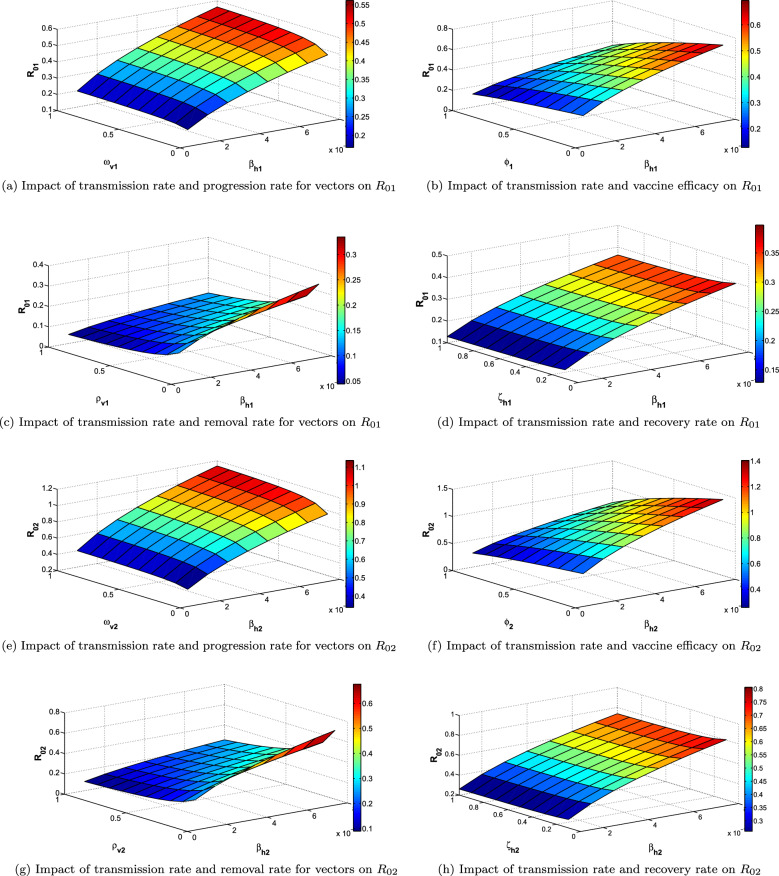
Surface plots to show the impact of different parameters involved in reproduction numbers.

Sensitivity analysis is carried out in this section to analyse the influence of the different parameters involved in the reproduction numbers of model 4. We employed the PRCC techniques separately for both reproduction number to show the role of the parameters in the reproduction number. It can be observed from Fig. 3a,b, that transmission rates for human as well as vectors, vaccination rates and vaccine efficacy are very sensitive to the reproduction number. To be more specific transmission rates are positively correlated with the reproduction numbers. It is observed in the Fig. 4a–h that, with the increment in the transmission rates from vectors to human, the reproduction number is also increased. Vaccine efficacy is negatively correlated with respect to the reproduction number. In the Fig. 4b,f, it can be seen easily that increment in the vaccine efficacy lower the reproduction number which means we can control the disease by introducing the vaccines that have stronger efficacy. The Fig. 4c,g describe the behaviour of reproduction numbers for both of the disease depending upon the transmission rate and removal of vectors. It is shown that we can also control the reproduction number and hence the disease by removing the more vectors from the environment. On the similar fashion, the Fig. 4a,e describes the dependence of reproduction numbers upon the transmission rate and progression rates. It can be seen that progression rate is positively correlated with respect to the reproduction number. Similarly, the other parameters involved in reproduction number like progression rates, recovery rates and quarantine measures have also great impact on reproduction numbers. We also presented the pie charts for both of the reproduction numbers in Fig. 3c,d that gives the percentage influence of all the parameters.

### Numerical assessment

**Figure 5:**
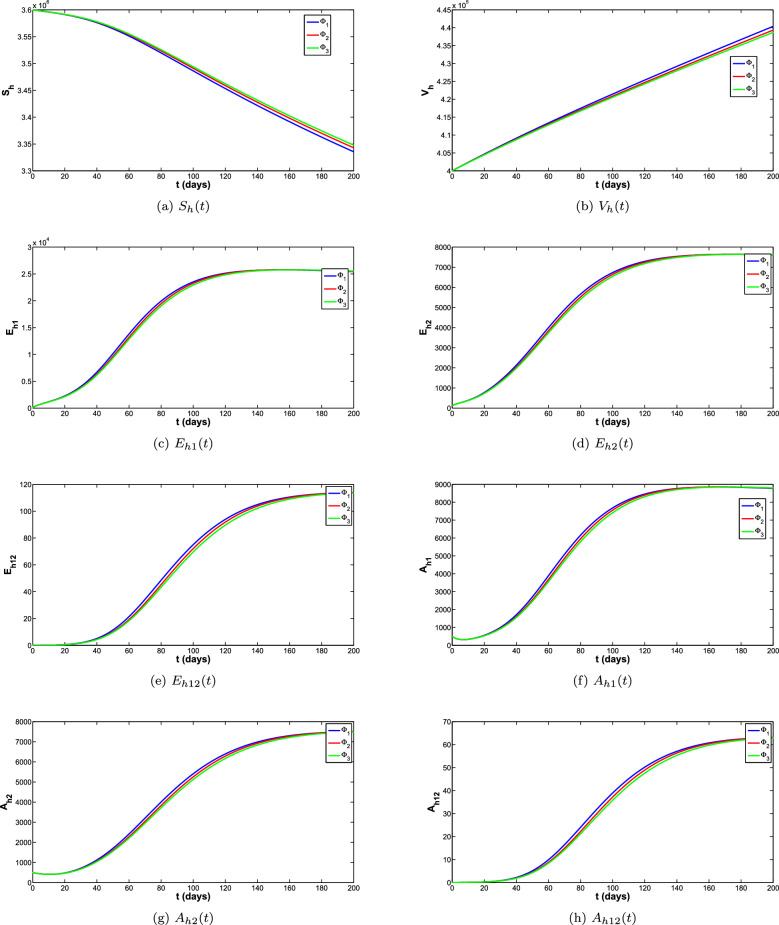
Comparison of all classes for different denominator functions.

To obtain the correct long-time behaviour of the model with NSFD, some denominator functions available in the literature were explored. The impact of these denominator functions for all the compartments of the model is shown in the Fig. 5a–f. The different denominator functions are $\Phi _1=h^{\sigma }$, $\Phi _2=\frac{h^{1-\sigma }(1-E_{\sigma }(-(\mu _h^{\sigma } p)^{\sigma }))}{(E_{\sigma }(-(\mu _h^{\sigma } p)^{\sigma }))\Gamma (2-\sigma )\mu _h^{\sigma }}$, $\Phi =\frac{e^{\mu _h^{\sigma }}-1}{\mu _h^{\sigma }}$ are used for the simulations. The reproduction number is the quantity that plays an important role for the disease to die out or spread. Different scenarios for the reproduction number of both diseases are listed below and for these scenarios different simulations are carried out shown in the Fig. 8a–d. These simulations are showing that when reproduction number is greater than 1 the disease is spreading and when reproduction number is less than 1 the disease eventually die out.

Different scenarios are considered: Scenario-1: ${\mathcal {R}}_{01} \ge 1, {\mathcal {R}}_{02} \ge 1$, Scenario-2: ${\mathcal {R}}_{01} \le 1, {\mathcal {R}}_{02} \ge 1$, Scenario-3: ${\mathcal {R}}_{01} \ge 1, {\mathcal {R}}_{02} \le 1$ and Scenario-4: ${\mathcal {R}}_{01} \le 1, {\mathcal {R}}_{02} \le 1$.

In Fig. 9a–d, the epidemiological affect of quarantine measure is assessed. It can be observed that the quarantine measure has great impact in averting new dengue strains infections, Specifically, maximum number of cases averted is recorded when quarantine rates are as much as $\eta _{i1}=0.05$, $\eta _{i1}=0.10$ and $\eta _{i1}=0.20$.

In Fig. 10a–c, simulations of the infected compartments are presented when vaccine parameters $\psi$ and $\phi _1$ are varied. It is interesting that maximum number in the dengue strain-1 infection averted is recorded when $\psi =0.20$ and $\phi _1=0.85$. Similar conclusion can be reached for strain-2 for maximum number dengue strain-2 infection averted and this accounts when $\psi =0.20$ and $\phi _2=0.85$ in Fig. 10d–f. Hence to keep the co-circulation of both dengue strain as low as possible vaccination rate must be stepped up to 0.20 per day while keeping effectiveness of vaccine against strain-1 and strain-2 infections at 0.85.

The phase portraits of the exposed, Asymptomatic infected and Symptomatic infected at different initial conditions and for different cases of reproduction numbers are presented in Fig. 11a–f, respectively. In Fig. 11a–c it can be observed that the solution paths for all the infected classes (Exposed, Asymptomatic and Symptomatic infected) tend towards the infection free equilibrium when reproduction number is less than 1 irrespective of the initial conditions and order of the derivative. Similarly it can also be observed in Fig. 11d–f that the solution paths for all the infected classes tend towards the endemic equilibrium when reproduction number is greater than 1 irrespective of the initial conditions and order of the derivative.

**Figure 6:**
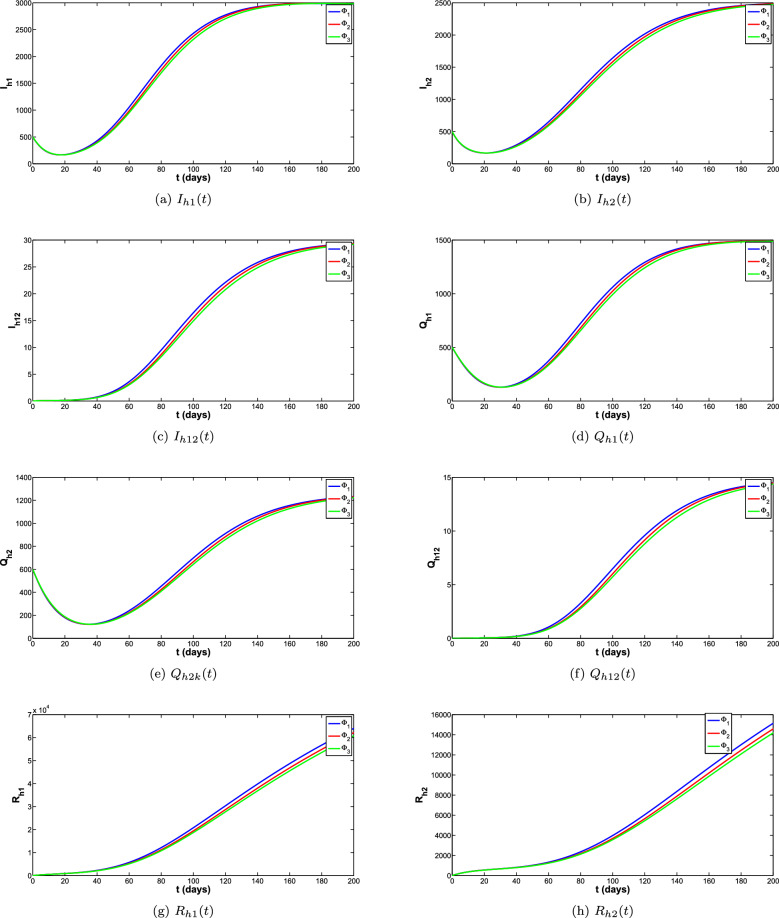
Comparison of all classes for different denominator functions.

**Figure 7:**
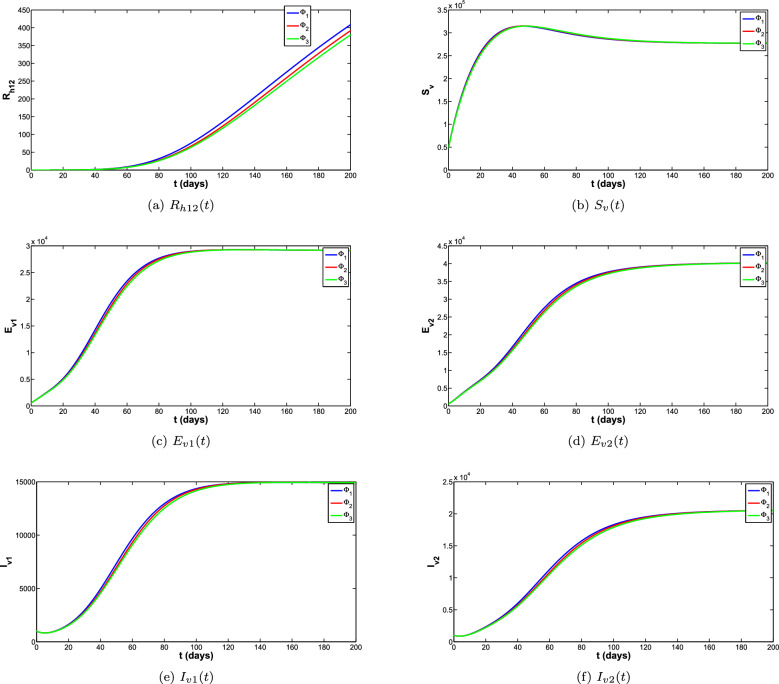
Comparison of all classes for different denominator functions.

**Figure 8:**
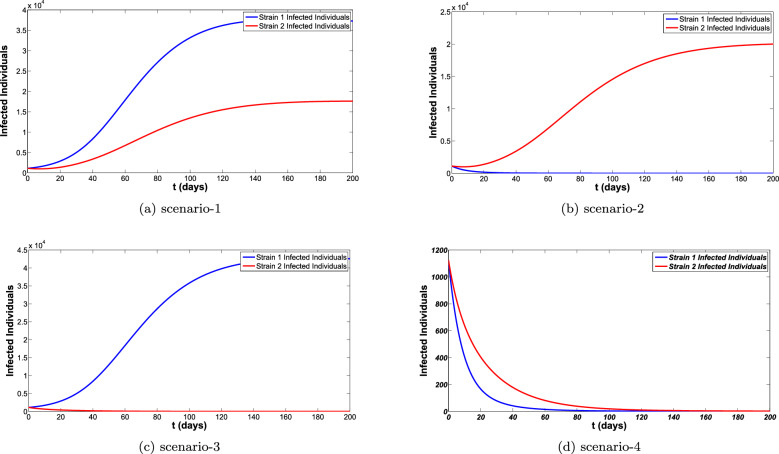
Comparison of infected individuals for different scenarios of reproduction number.

**Figure 9:**
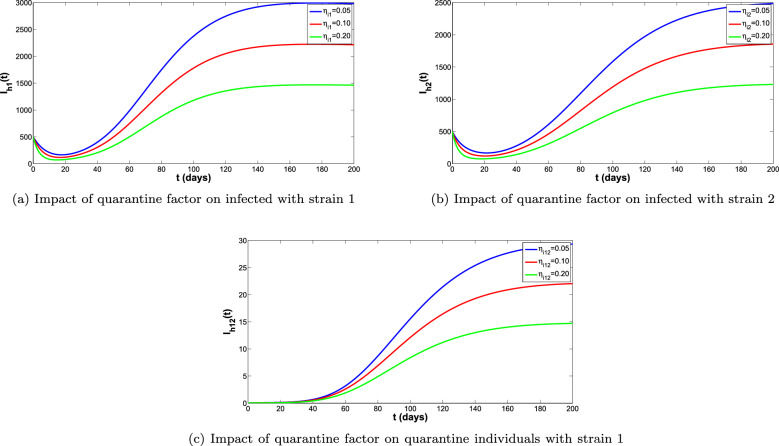
Impact of quarantine measures on infected and quarantine individuals.

**Figure 10:**
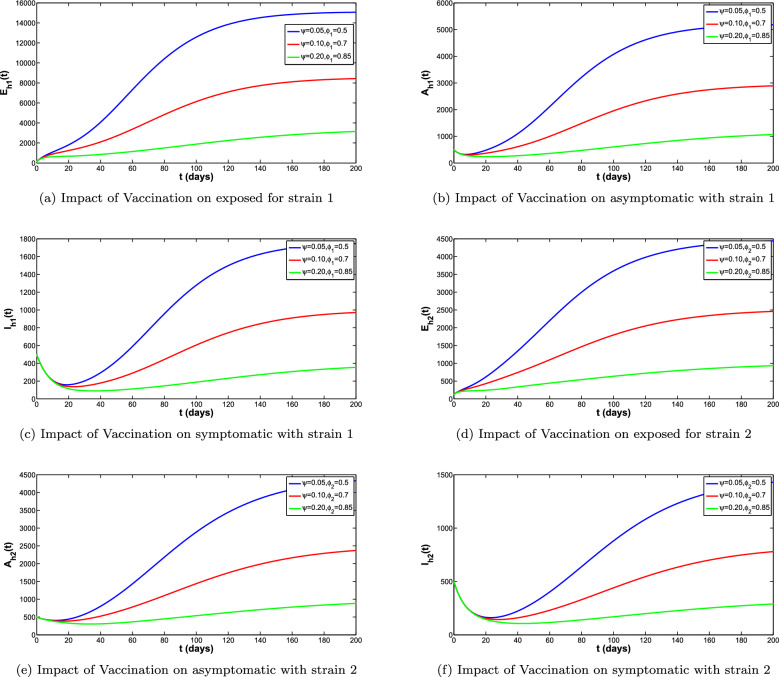
Impact of vaccination and vaccine efficacy on infected compartments.

**Figure 11:**
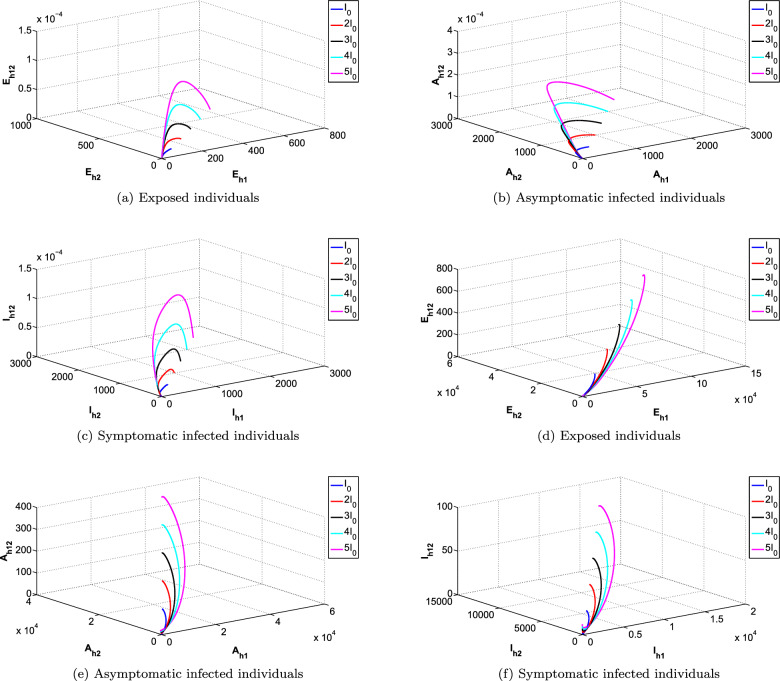
Simulations of infected classes separately with co-infection at different initial conditions.

## Conclusion

In this paper, a comprehensive mathematical model is proposed for two strains of dengue virus with saturated incidence rates and quarantine measures. Imperfect dengue vaccination is also assumed in the model. Existence, uniqueness and stability of the new model are established using some results from fixed point, measure theory and degree theory. Additionally, well constructed Lyapunov function candidates are also applied to prove the global stability of infection-free and endemic equilibria. It is also demonstrated that the model system is generalized Ulam–Hyers stable under certain appropriate conditions. The model is fitted to the real data for dengue epidemic for the city of Espirito Santo in Brazil. For the approximate solution of the model, a non-standard finite difference(NSFD) approach is applied. The behaviour of the NSFD is also assessed under different denominator functions and it is observed that the choice of the denominator function could influence the solution trajectories. Different scenario analysis are also assessed when the reproduction number is below or above one. Furthermore, simulations are also presented to assess the epidemiological impact of dengue vaccination and quarantine measures for infected individuals.Some of the major highlights of the qualitative analysis are as follows:

(i) The strain 1 and strain 2 sub-models are qualitatively analyzed, investigating the stability in the sense of Lyapunov which are presented in “Analysis of the sub-models”.
(ii) The full model’s infection-free equilibrium is proved to be locally stable, as presented in Theorem 4.2.
(iii) Existence, uniqueness and stability of the complete model are presented in “Existence, uniqueness and Ulam–Hyers stability of the complete model” with the help of results from fixed point theory and degree theory.

The major highlights of the numerical analysis which are carried out using the non-standard finite difference scheme and are given in “Nonstandard finite difference scheme” are presented below:

(i) The model is fitted to the real data for the city of Espirito Santo in Brazil.
(ii) The choice of the denominator function influences the behaviour of the solution under consideration.
(iii) Sensitivity analysis of the reproduction number for both strains are carried out to show the sensitive parameters and the result is shown with the help of pie chart,
(iv) The solution profiles when the reproduction numbers of both strains are either below or greater than one as well as when one reproduction number dominates the other, are also investigated.
(v) Different scenario analyses to investigate the epidemiological impact of dengue vaccination and quarantine for infected individuals shows that these two measures could greatly reduce the co-spread of both strains within a population.

The research in this paper can be extended in the following ways: One could consider stochastic equivalence as well as fractal fractional form of the current model for a possible research problem. Approximate solution of the model using some other novel numerical schemes that can yield the better results can also be considered. Moreover, one could also establish the existence, uniqueness and stability results using some novel fixed point theorems

## Data Availability

The datasets used and/or analysed during the current study available from the corresponding author on reasonable request.
